# Fine‐needle aspiration as an alternative to core needle biopsy for tumour molecular profiling in precision oncology: prospective comparative study of next‐generation sequencing in cancer patients included in the SHIVA02 trial

**DOI:** 10.1002/1878-0261.12776

**Published:** 2020-09-15

**Authors:** Célia Dupain, Julien Masliah‐Planchon, Céline Gu, Elodie Girard, Pierre Gestraud, Pauline Du Rusquec, Edith Borcoman, Diana Bello, Francesco Ricci, Ségolène Hescot, Marie‐Paule Sablin, Patricia Tresca, Alexandre de Moura, Delphine Loirat, Maxime Frelaut, Anne Vincent‐Salomon, Charlotte Lecerf, Céline Callens, Samantha Antonio, Coralie Franck, Odette Mariani, Ivan Bièche, Maud Kamal, Christophe Le Tourneau, Vincent Servois

**Affiliations:** ^1^ Department of Drug Development and Innovation (D3i) Institut Curie Paris & Saint‐Cloud France; ^2^ Department of Genetics Institut Curie PSL Research University Paris France; ^3^ Department of Pathology Institut Curie PSL Research University Paris France; ^4^ INSERM U900 Research Unit Institut Curie Saint‐Cloud France; ^5^ INSERM U1016 Faculty of Pharmaceutical and Biological Sciences Paris Descartes University Paris France; ^6^ Paris‐Saclay University Paris France; ^7^ Department of Radiology Institut Curie PSL Research University Paris & Saint‐Cloud France

**Keywords:** core biopsy, fine‐needle aspiration, next‐generation sequencing, precision medicine, SHIVA02 trial, tumour molecular profiling

## Abstract

High‐throughput molecular profiling of solid tumours using core needle biopsies (CNB) allows the identification of actionable molecular alterations, with around 70% success rate. Although several studies have demonstrated the utility of small biopsy specimens for molecular testing, there remains debate as to the sensitivity of the less invasive fine‐needle aspiration (FNA) compared to CNB to detect molecular alterations. We aimed to prospectively evaluate the potential of FNA to detect such alterations in various tumour types as compared to CNB in cancer patients included in the SHIVA02 trial. An in‐house amplicon‐based targeted sequencing panel (Illumina TSCA 99.3 kb panel covering 87 genes) was used to identify pathogenic variants and gene copy number variations (CNV) in concomitant CNB and FNA samples obtained from 61 patients enrolled in the SHIVA02 trial (NCT03084757). The main tumour types analysed were breast (38%), colon (15%), pancreas (11%), followed by cervix and stomach (7% each). We report 123 molecular alterations (85 variants, 23 amplifications and 15 homozygous deletions) among which 98 (80%) were concordant between CNB and FNA. The remaining discordances were mainly related to deletions status, yet undetected alterations were not exclusively specific to FNA. Comparative analysis of molecular alterations in CNB and FNA showed high concordance in terms of variants as well as CNVs identified. We conclude FNA could therefore be used in routine diagnostics workflow and clinical trials for tumour molecular profiling with the advantages of being minimally invasive and preserve tissue material needed for diagnostic, prognostic or theranostic purposes.

## Graphical abstract

High‐throughput DNA analyses in cancers allow the detection of molecular abnormalities that can guide patients' treatment. The collection of a tumour DNA is mainly performed on a biopsy, an invasive procedure for the patients. Another less invasive method consists in using a fine needle. We showed that fine‐needle biopsies are as suitable as a classical biopsy for DNA analysis.

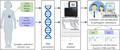

**Abbreviations**
CNB: core needle biopsy
CNV: copy number variation
CT: computerized tomography
EDTA: ethylenediaminetetraacetic acid
FDR: false detection rate
FNA: fine‐needle aspiration
G: Gauge
NGS: next‐generation sequencing
QC: quality control
RPMI: Roswell Park Memorial Institute
SNV: single nucleotide variation
TSCA: Truseq Custom Amplicon

## 1 Introduction

With the large deployment of next‐generation sequencing (NGS) and the increasing knowledge on cancer genomics goes an accelerated demand for molecular analyses.

Precision medicine clinical trials in oncology, based on molecular analyses to prevent, diagnose and treat patients, use image‐guided biopsies as the gold standard starting material to perform these analyses [1, 2, 3]. However, tumour inaccessibility and noncontributive samples limit the number of patients screened in these trials, which can already be selective regarding inclusion (e.g., previous treatments, tumour type, age). An analysis showed that among four clinical trials conducted at the National Cancer Institute's (NCI), 26% of samples collected did not meet the quality control (QC) criteria required in the trials (i.e., insufficient material, low tumour cellularity, poor DNA quality and/or quantity). For trials requiring sequential biopsies (i.e., paired predose and postdose), this rate increases up to 50% [4]. This number is in correlation with failure rates observed in other precision medicine clinical trials, such as the SHIVA01 trial (27% failure rate), MOSCATO (21%), and WINTHER (38%) [1, 2, 3]. In addition, the invasive character of the procedure for the patients highlights the necessity to provide other alternative sample types to perform molecular profiling.

Most of the samplings are ultrasound (US) or computed tomography (CT)‐guided and performed by interventional radiologists, but there are no conventional guidelines regarding the procedure. Core needle biopsy (CNB) represents the gold standard for tumour samplings and is performed using semi‐automatic or automatic cutting needles of 18 or 16 Gauge (G) calibres to obtain tumour fragment. When the lesion is inaccessible to biopsy, a fine‐needle aspiration (FNA) may be feasible percutaneously or endoscopically [5, 6]. FNA is a minimally invasive sampling technique that can be performed on an outpatient basis and generally requires needles of 25–20 Gauge. Most cytological preparations are frozen which provides better quality results for molecular analysis [7, 8]. FNA is the reference sampling technique for some cancers (e.g., thyroid nodule, uveal melanoma) [9, 10]. Similarly, in paediatric tumours where tumours can be difficult to access, FNA is more and more used as it allows a quick, minimally invasive and accurate diagnosis [11]. In a large series of 774 patients, FNA guided by endo‐bronchial echo‐endoscopy allowed to determine the non‐small‐cell histological subtype of lung cancer in 77% of cases and to carry out a screening of *EGFR* mutations in 90% of the cases [12].

Despite its advantages, FNA remains underused in most radiological teams for molecular screening, based on the common principle that a larger size of sample is correlated to a better molecular analysis [13], and due to the lack of common validated preparation protocol [14, 15]. Of note, CNB remains the gold standard for diagnosis since the morphology of the tumour is essential to establish the histological diagnosis. Hence, it is necessary to determine the reliability of tumoral genomic analyses on FNA to screen prognostic and predictive biomarkers.

Several studies have begun to address this question in specific tumour types. Gleeson *et al*. [16] successfully detected characteristic pathogenic variants and prognosis biomarkers by NGS on 102 FNA samples from rectal cancer. In papillary thyroid carcinoma, Yu *et al*. [17] even demonstrated a higher sensitivity of NGS analysis in FNA compared to conventional methods for mutation detection. Finally, in lung cancer, several studies also confirm the feasibility of variants detection from FNA samples [18, 19, 20, 21]. Yet, molecular analyses obtained from FNA in tumours of various histology are rare and only few studies assessed the comparison with concomitant CNB samples [14, 22, 23, 24]. All studies suggest FNA is a valuable source of material for molecular testing, and Roy‐Chowdhuri *et al*. [14] showed that FNA presented advantages regarding cellularity, tumour fraction and a higher coverage as compared to CNB.

To our knowledge, comparison of genomic analyses, comprising both mutations and copy number alterations, between concomitant CNB and FNA on various tumour types has not been studied so far. The aim of this study was to assess if FNA samples could be a reliable material for genomic analyses, in order to investigate prognostic/theranostic biomarkers in real time, in different solid tumour types. For this, we performed concomitant CNB and FNA in patients enrolled in the SHIVA02 trial followed by targeted NGS on tumour DNA.

## 2 Materials and methods

### 2.1 Patient enrolment

SHIVA02 (Evaluation of the Efficacy of Targeted Therapy Based on Tumour Molecular Profiling in Patients With Advanced Cancer Using Each Patient as Its Own Control; ClinicalTrials.gov identifier: NCT03084757) is a multicentric open‐label nonrandomized controlled phase II trial sponsored by the Curie Institute. All patients were included after written informed consent. Patients older than 18 years with any type of recurrent and/or metastatic cancer who failed standard therapy were eligible for the study provided their disease was measurable and accessible for a biopsy or resection of a metastatic site.

The study methodologies conformed to the standards set by the Declaration of Helsinki. The trial was approved by the ethics committee and the French ‘Agence Nationale de Sécurité du Médicament et des produits de santé’.

Funding sources played no role in study design, collection, analysis and interpretation of data; in the writing of the report; and in the decision to submit the article for publication.

### 2.2 Tumour sample collection and processing

This research was conducted within the frame of the SHIVA02 protocol, as an ancillary study in order to answer to secondary objectives aiming to assess the reliability of FNA for molecular analyses. All other decisions (diagnosis, results of molecular analyses and Molecular Biology Board decisions) were based on CNB analyses only.

Sampling was performed by interventional radiologist using ultrasound or computed tomography guidance with a coaxial technique. The guide needle (19 or 17 G) was placed in the periphery of the target lesion. FNA sampling with a 22 G needle (2 punctures) was performed at first. The aspiration product was collected in a vial containing RPMI‐EDTA medium. CNB samples (4 samples) were secondly obtained with a semi‐automatic cutting needle (18 or 16 G) (Fig. S1). Both CNB and FNA samples were taken from the same area, in the centre or the tumour. Biopsy specimens were placed in a vial containing RPMI medium. All specimens were transferred to the pathology department within 1 h.

Samples from CNB were frozen in liquid nitrogen, and quality QC (tissue quality and tumour cellularity) was determined from cryosections after H&S staining. For FNA, samples were split into two parts, one being used for QC and the other for DNA extraction. For QC, 100–300 µL (depending on the viscosity of the liquid) from the suspension were centrifugated 5 min at 700 r.p.m. onto slides using CytoSpin™ (Thermo Scientific, Waltham, MA, USA). Then, Diff Quick™ (Microptic, Barcelona, Spain) staining was used for immediate assessment of cellularity by a pathologist. The remaining FNA sample was centrifugated 10 min at 1500 ***g***, and the pellet was collected for DNA extraction.

**Fig. 1:**
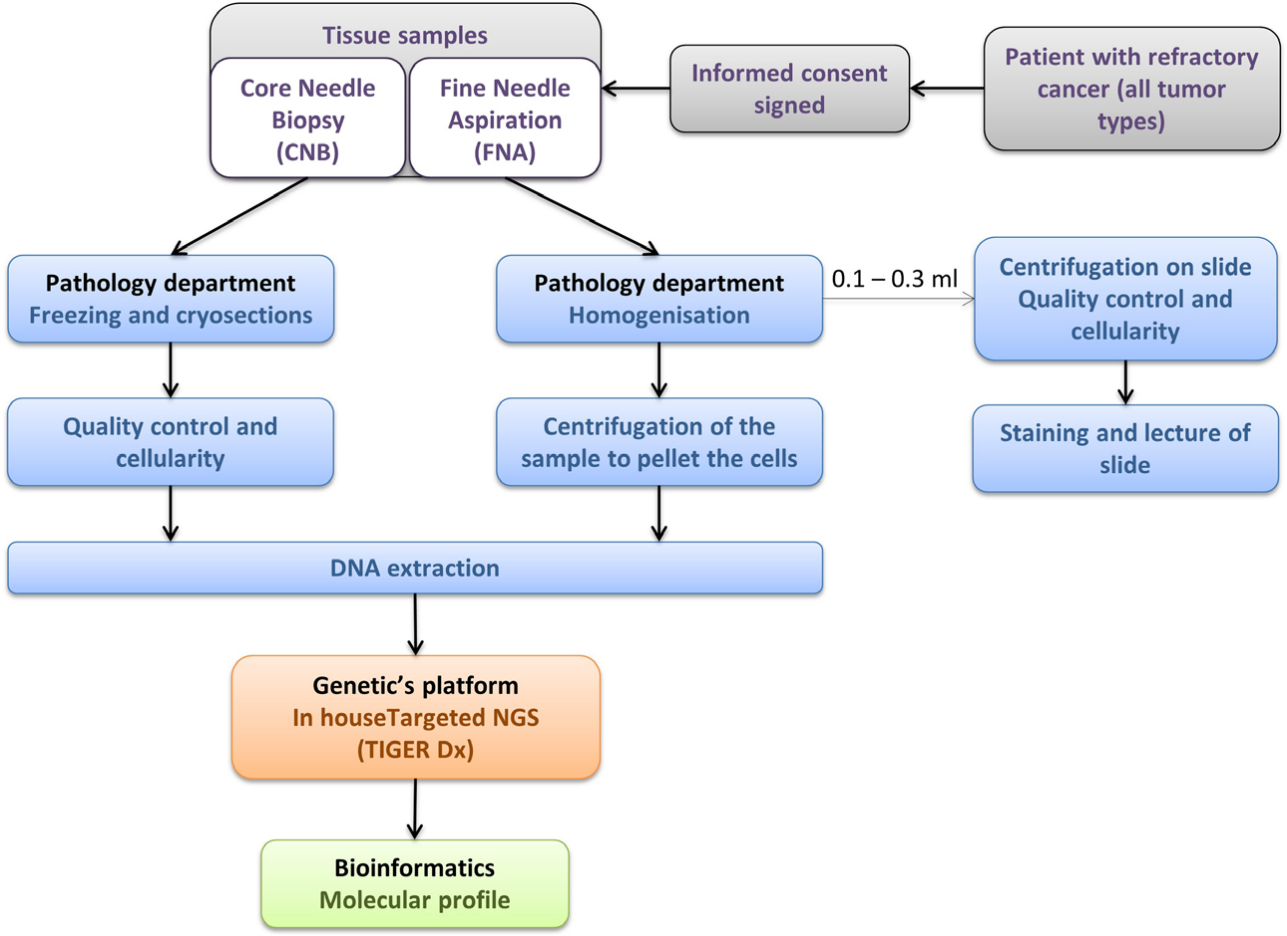
Sample analysis workflow.

For CNB, the estimation of the neoplastic cell percentage was evaluated through eye‐balling of the slides, as the percentage of neoplastic cells *versus* all cells in the stroma, and was made as accurately as possible in decile. For FNA, the number of neoplastic cells was estimated over the surface of the cytospot obtained after CytoSpin™ centrifugation. The cell fraction was evaluated by a cytopathologist of the department, randomly chosen, to evaluate either CNB or FNA, in the frame of the clinical routine diagnosis. The global workflow of samples is presented in Fig. 1.

### 2.3 DNA extraction and library preparation

DNA was extracted from concomitant CNB and FNA pair samples with a tumour cell fraction ≥ 10%.

DNA from CNB samples and FNA samples pellets were extracted by the Biological Resource Center of Institut Curie, using Phenol : Chloroform:Isoamyl Alcohol (Invitrogen, Carlsbad, CA, USA) under manufacturer's instructions.

DNA samples were quantified by spectrophotometer (NanoDrop™ ND‐2000; Thermo Scientific) and Qubit™ (Life Technologies, Carlsbad, CA, USA), and integrity was assessed by migration on agarose gel, before storage at −20 °C. Then, 100 ng of DNA was used to prepare the library of an in‐house NGS‐based gene panel (99.3 kb, 1504 amplicons) covering 87 genes using the TruSeq Custom Amplicon Low Input library prep kit (Illumina, San Diego, CA, USA) under manufacturer's recommendations.

### 2.4 NGS‐based gene panel sequencing and analysis pipeline

Libraries were sequenced on the NextSeq500 (Illumina). This resulted in the production of two barcodes of 75 bp paired‐end reads per sample, called respectively panels ‘A’ and ‘B’ to target both DNA strands at all loci. In this study, alterations found by both panels were considered, but only the values obtained with panel ‘A’ were reported regarding allele frequency and copy number.

For variant calling, copy number alteration detection and quality control, reads were mapped using bowtie2 (v2.2.5) [25] on the human reference genome (hg19 assembly) using the following parameters: 1 mismatch allowed in the 22 bp‐seed, 1 alignment reported per read. Alignment files were then intersected on the targeted regions using bedtools (v2.21.0) [26], and resulting singleton alignments were discarded using samtools (v0.1.19) [27].

Aligned pairs were finally assigned to a specific amplicon, and sequenced primers were trimmed using a homemade python (v2.7.9; Python Software Foundation, Wilmington, DE, USA) script and the pysam module (v0.9.1.4). Amplicon assignment was performed when both first read (5′) and second read (3′) matched the amplicon start and end, given a tolerance of ±3 bp on each extremity. At this step, reads resulting from the fusion of several adjacent amplicons were excluded and raw counts per amplicon (number of assigned pairs) were computed for the copy number alteration strategy.

### 2.5 Copy number alterations

Copy number alterations were called using the combination of homemade r (v3.2.0) scripts and ioncopy amplicon call CRAN package [28]. Raw counts were first normalized by the median over all the amplicons per barcode and then by the median over all barcodes per amplicon. Amplicons showing inconsistency during pilot study were discarded from further analysis.

In parallel, segmentation of the log2 ratio per coordinated‐ordered amplicons was performed using CRAN cghseg package [29]. CNV statuses were defined depending on the log2 median normalized value of the segment which corresponds to < −1 and > 2 for homozygous deletions and amplifications, respectively.

### 2.6 Variant calling

Variant calling of both single nucleotide variations (SNVs) and small insertion/deletions (indels) was performed on the processed alignment files using a combination of the mpileup module of samtools (taking into account anomalous read pairs without base quality recalibration, considering a minimum mapping quality of 0 and base quality of 17, and a maximum read depth of 1 M) and varscan2 (v2.4.1) [30]. Variants were reported if the number of reads supporting the alternative allele was superior or equal to 1, at a locus covered by at least 30 reads, and if the allelic ratio of this variant was superior or equal to 1%.

A dedicated strategy using Amplicon Indel Hunter (v1.1.0) [31], with default parameters, was applied to call intermediate‐size indels (> 10 bp).

Several files were computed per barcode in order to assess the run overall quality.

Sequencing and target enrichment quality were controlled using several metrics and affiliated warnings: (a) number of sequenced reads (warning if below 1 M), (b) percentage of aligned reads, and (c) percentage of aligned and processed reads (after the amplicon assignment). Moreover, each barcode was annotated with the percentage of the coding regions of the target covered by at least 100X, 300X and 1000X depth.

All data obtained after bioinformatics analyses will be referred to as ‘Total data’ in the following sections.

### 2.7 Validation of detected alterations

Total data were then validated using the algorithm described under this section, to identify pathogenic alterations.

Variants were classified into five classes: Pathogenic (known hotspot variants); Likely pathogenic (based on *in silico* analysis); Uncertain significance; Likely benign (probably not pathogenic based on *in silico* analysis); and Benign (other variants certainly not pathogenic).

For SNV, missense and in‐frame insertion/deletions hotspot variations were taken into consideration. Missense hotspot mutations were selected based on the literature, the prediction databases PolyPhen‐2 and SIFT, COSMIC public tumour database, cBioPortal, local variant database and based on the localization of the variation within the gene (i.e., variant in a functional domain). In‐frame insertion/deletions were kept if described in public tumour database (COSMIC, Tumor portal, cBioPortal and Cancer Hotspot) or in the literature. The allelic frequency considered for variant was ≥ 5%. Variants not considered as activating in oncogenes were polymorphisms with a frequency > 0.1% in 1000 genome (08/2015 version) and/or ESV (ESP6500), synonymous, nonsense mutations and frameshift insertion/deletions.

For tumour suppressor genes, bi‐allelic alterations were considered as inactivating and comprised homozygous inactivating alterations; composite heterozygous inactivating alterations; and inactivating mutations combined with loss of heterozygosity.

Mutations considered as inactivating were nonsense mutations, frameshift insertion/deletion, splicing mutations, intragenic large deletions or duplications, and missense mutations already described in the literature as inactivating.

Variants not considered as inactivating were polymorphisms with a frequency > 0.1% in 1000 genome and/or ESV; silents without of the first/last 2 bp of the exons (since they can alter the splicing); and unknown inactivating missense mutations.

Data obtained after validation and selection of relevant molecular alterations according to the criteria described above will be referred to as ‘Validated data’ in the following sections.

### 2.8 Statistics

Correlation between frequencies of pathogenic variants of FNA and CNB was computed by taking the transformed value asin(√(frequency)) and using the Pearson's correlation coefficient. For CNV alterations, the correlation between pairs of samples was assessed by taking the log ratios of overlapping segments and computing Pearson's correlation coefficient with associated *P*‐value. Multiple testing was controlled with the Benjamini–Hochberg procedure.

By using graphpad prism (GraphPad Software, San Diego, CA, USA) 4 software, Mann–Whitney test was used to compare the group of CNB samples *versus* FNA samples regarding: tumour cell content, DNA concentrations, DNA ratios (both 260/280 and 260/230), reads number, the percentage of mapped reads, the percentage of mapped reads on target, and the percentage of depth ≥ 100.

Kruskal–Wallis test followed by Dunn's test was used to compare CNB and FNA samples' DNA concentrations within the three groups of concentration ranges.

*P* < 0.05 was considered as statistically significant. *P < 0.05; **P < 0.01; ***P < 0.001.

## 3 Results

### 3.1 Patients' description

**Fig. 2:**
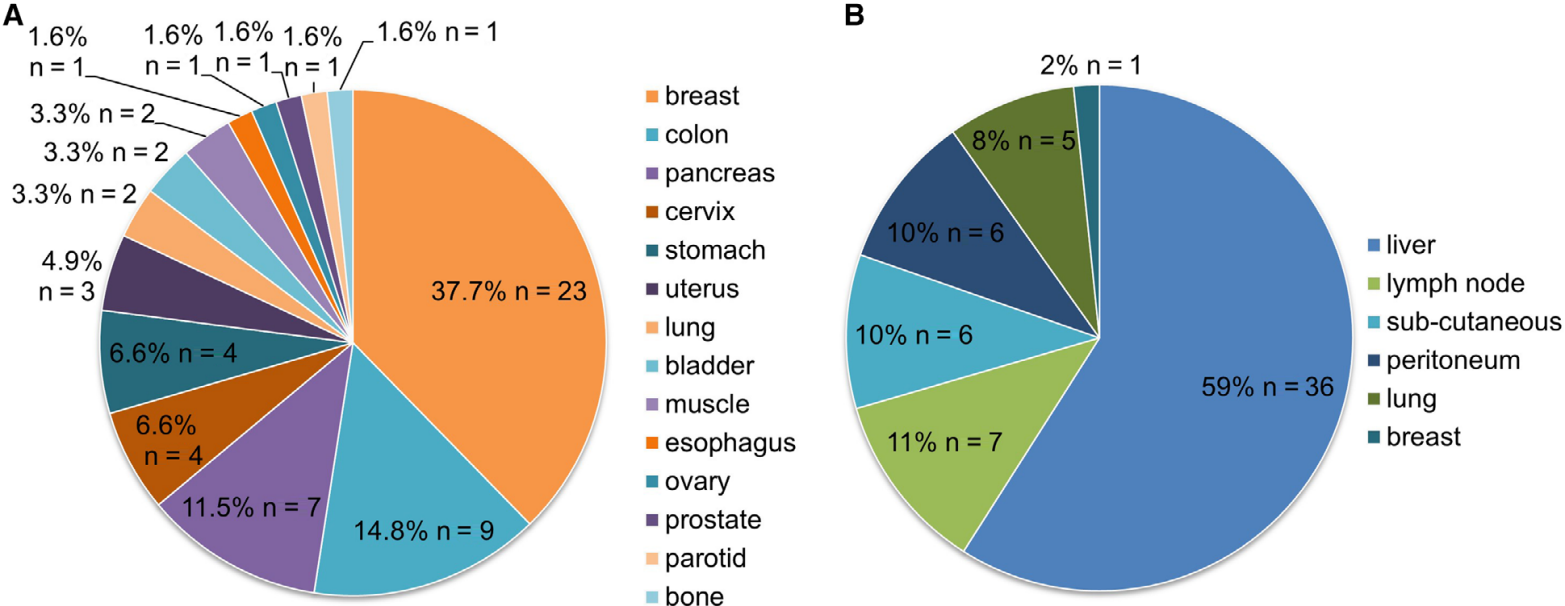
Distribution of cancer types (A) and biopsy sites (B) of the 61 patients screened.

CNB and FNA samples from 61 patients enrolled in the SHIVA02 trial were analysed corresponding to 16 different tumour types (Fig. 2 and Table S1). Main tumour types were breast (38%, *n* = 23), colon (15%, *n* = 9) and pancreas (11%, *n* = 7), followed by cervix and stomach (7% each, *n* = 4).

Concomitant CNB and FNA were performed mainly on metastases, in *n* = 61 patients from six different localizations (i.e., liver, lymph node, subcutaneous, peritoneum, lung and breast). The large majority of cases were obtained in liver (57%, *n* = 36), followed by lymph nodes (12%, *n* = 7) and subcutaneous locations (12%, *n* = 6).

### 3.2 Both CNB and FNA samples showed no difference in DNA and NGS qualities

Regarding cellularity, the average tumour cell content was statistically lower in FNA compared to CNB (31% in FNA *versus* 52% in CNB), Table S1 and Fig. S2). The average concentration of DNA was similar between CNB [122.1 ng·µL^−1^ (0–361 ng·µL^−1^)] and FNA [147.8 ng·µL^−1^ (2.9–414 ng·µL^−1^)]. The large majority of CNB and FNA samples had a DNA concentration > 100 ng·µL^−1^ (*n* = 36 CNB and *n* = 37 FNA; Table S1 and Fig. S3), and all samples had the minimum quantity of DNA needed for NGS analysis (i.e., 100 ng). DNA quality also showed no statistical difference between the two specimens with the same average 260/280 and 260/230 ratios (1.9 and 2 respectively, Table S1).The large majority of samples also showed no degradation after migration on agarose gel (Table S1), independently if it came from CNB or FNA.

Regarding NGS analysis, the average read number was 11 426 798 [range (648 474–22 164 826)] in CNB samples *versus* 11 240 452 [range (1 227 758–21 584 380)] in FNA samples. The mean percentage of mapped reads was equal to 81.78% (1.46–98.63) for CNB and 83.52% for FNA (12.60–98.37). Finally, when considering a depth ≥ 100X , the coverage was of 93.11% (3.88–98.29) for CNB and 95.43% (76.53–98.22) for FNA (Table S1).

No statistical difference was found between CNB and FNA samples regarding DNA concentrations, DNA ratios (both 260/280 and 260/230), reads number, percentage of mapped reads, percentage of mapped reads on target, and percentage of depth ≥ 100X. The percentage of mapped reads and the depth of coverage were both significantly correlated and similar between CNB and FNA samples (*r* = 0.831 and *r* = 0.815 respectively).

### 3.3 Relevant validated molecular alterations are detected in both CNB and FNA from the same pair at high rates

**Fig. 3:**
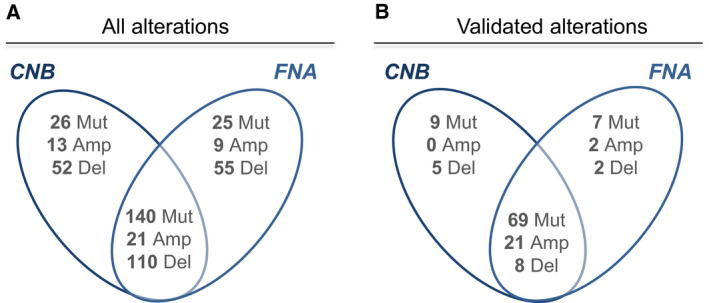
Alterations common and specific to CNB and FNA, before and after validation by the biologists. For mutations within tumour suppressor genes, only homozygous mutations are considered, except in cases where no mutation is detected in a sample from a pair, and an heterozygous mutation is detected in the other sample from the same pair. Mut, pathogenic SNV or Indel; Amp, amplification; Del, homozygous deletion.

Total data from NGS analysis revealed 191 variants and 260 copy number variations (CNV). Among the CNV, 17% (*n* = 43) were amplifications and 83% (*n* = 217) were deletions (Fig. 3A).

Seventy‐three per cent of pathogenic variants (140/191), 49% (21/43) of amplifications and 51% (110/217) of deletions were detected in both CNB and FNA (Fig. 3A). Remarkably, the number of variants detected per patient was similar in both the corresponding CNB and FNA samples (Fig. S4).

**Fig. 4:**
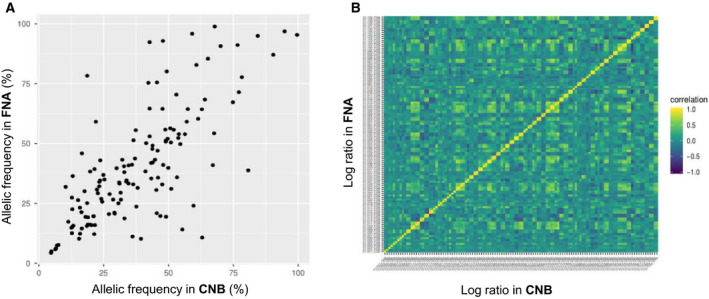
Correlations of variant frequency (A) and CNV log ratio (B) between CNB and FNA samples.

A high correlation between CNB and FNA regarding pathogenic variants and CNV alterations was observed. For pathogenic variants, the correlation of allelic frequencies was 0.743; for CNV alterations, 90% of pairs of FNA and CNB showed a significant correlation of copy number profiles at false detection rate (FDR) 5% (Fig. 4A,B).

After validation to identify pathogenic variants from the total data (‘validated data’), 85 pathogenic variants and 38 CNV alterations (*n* = 23 amplifications and *n* = 15 homozygous deletions) were subsequently retained as clinically relevant (Fig. 3B and Table S2). Among them, the rate of alterations detected in both CNB and FNA from the same pair increases compared to total data: 81% (69/85) pathogenic variants were commonly detected in pairs, 91% (21/23) of amplifications and 53% (8/15) of homozygous deletions (‘concordant’ alterations, Fig. 3B). In total, 98 (80%) validated pathogenic alterations were concordant between CNB and FNA. *TP53* was the most frequently altered gene (32 alterations), followed by *PIK3CA* (11 alterations), *KRAS*, and *ESR1* (11 alterations each) and *CDKN2A/CDKN2B* (8 alterations) (Table S2). Interestingly, 71% of patients (43/61) showed no discordant molecular result between CNB and FNA samples after NGS analysis (Table S2).

### 3.4 Analysis of both CNB and FNA samples successfully identified actionable targets

Actionable molecular alterations represented 57% (70/123) of all validated alterations. Among them, 76% (53/70) were concordant between CNB and FNA *versus* 13% (16/123) of discordant actionable molecular targets (Table 1 and Table S1). Discordances concerned 14 patients and were mainly related to CNV, particularly to heterozygous and homozygous deletions. Of note, the underevaluation of the alterations was not exclusively specific to FNA (7 alterations in FNA *versus* 9 for CNB, Table 1).

**Table 1:** Detailed description of validated and actionable alterations discordant between CNB or FNA.

Gene	CNB result	FNA result	Patient ID	Tumour location	Biopsy site
*CDKN2A*	Homozygous deletion	Homozygous deletion	19	Anal canal	Liver
*CDKN2A/B*	Homozygous deletion	Normal	10	Stomach	Liver
*CDKN2A/B*	Homozygous deletion	Homozygous deletion	16	Breast	Lung
*EGFR*	NA	Amplification	43	Cervix	Peritoneum
*FGFR1*	Gain	Amplification	3	Breast	Lymph node
*KRAS*	Heterozygous mutation	WT	9	Pancreas	Liver
*MAP2K4*	Homozygous deletion	Heterozygous deletion	49	Breast	Liver
*MAP2K4*	Homozygous mutation	Heterozygous mutation	55	Breast	Liver
*NF1*	Heterozygous mutation	Homozygous mutation	19	Anal canal	Liver
*PIK3CA*	Absent	Heterozygous mutation	5	Pancreas	Liver
*PIK3CA*	Heterozygous mutation	Absent	50	Colon	Liver
*PIK3R1*	Homozygous deletion	Heterozygous deletion	2	Ovary	Liver
*PIK3R1*	Homozygous mutation	Heterozygous mutation	46	Breast	Liver
*PTEN*	Heterozygous mutation	Homozygous mutation	6	Breast	Subcutaneous
*PTEN*	Normal	Homozygous deletion	27	Muscle	Peritoneum
*PTEN*	Homozygous deletion	Normal	46	Breast	Liver

## 4 Discussion

Biopsies are the gold standard material to establish a diagnosis and to perform molecular profiling in precision oncology [1, 2, 3, 32, 33]. Yet it is estimated that around 30% of advanced cancer patients remain unbiopsiable or samples fail to pass quality controls (i.e., insufficient material, low tumour cellularity, poor DNA quality and/or quantity) [1, 2, 34, 35, 36]. This restricts the access to precision medicine trials, highlighting the need to optimize other sample types for molecular profiling. Among the other sample types, liquid biopsies or cytology samples such a FNA are being more and more used in the clinical practice. However, although the least invasive procedure, liquid biopsies still have limits to their use in clinics as compared to FNA, as the circulating tumour DNA (ctDNA) extracted is polluted with constitutional DNA, tumour content cannot be estimated, histology stainings cannot be performed, and tumour heterogeneity cannot be studied.

In the current study, we prospectively analysed the relevant molecular alterations detected in concomitant CNB and FNA sample pairs obtained from a series of patients enrolled in the SHIVA02 trial, using an in‐house NGS‐based gene panel.

First, by comparing QCs related to CNB and FNA sample types, we observed that both DNA (concentration, ratios, quality) and NGS QCs (read number, mapped reads and depth of coverage) showed no difference between CNB and FNA, underlying their equivalent suitability for molecular assays. We noticed a difference regarding tumour cell content between CNB and FNA samples, which could be explained by the difference in references used for the evaluation of tumour cell percentage (i.e., over total cells including stroma for CNB *versus* cytospot surface for FNA). However, it is important to note that for our molecular analysis, cellularity did not affect the quality and quantity of DNA needed, or the quality of targeted NGS required in our study.

Molecular alterations were successfully detected by targeted NGS followed by data analysis, in both CNB and FNA sample types among patients. The data analysis consisted in a first step of bioinformatics analysis (so‐called ‘Total data’) and in a second step of validation of the Total data according to an algorithm (so‐called ‘Validated data’).

The bioinformatics analysis (Total data) showed a significant correlation between CNB and FNA regarding both the detection of pathogenic variants and CNV alterations. Seventy‐three per cent of pathogenic variants, 49% of amplifications and 51% of deletions were commonly detected in corresponding CNB and FNA pairs. Remarkably, after validation and selection of driver molecular alterations (validated data), the rate of common alterations detected between CNB and FNA from the same pair considerably increased up to 81% of pathogenic variants, 91% of amplifications and 53% of deletions. For pathogenic variants, this rate is in accordance with previous studies [14, 19, 24]. To our knowledge, no previous data exist on CNV. Our results highlight the leading role of a NGS data validation step, in order to take into account driver molecular alterations, and discard noise and irrelevant alterations (i.e., variants of uncertain significance). It is important to note that the remaining discordances between CNB and FNA did not depend on sample type (CNB or FNA) and mainly concerned deletions usually conditioned by tumour cellularity. We also showed that the discordances and success of molecular profiling did not depend on tumour type or biopsy localization.

Altogether, these observations underlie the equal reliability of both sample types for the identification of clinically relevant molecular alterations in tumour cells.

In this study, only metastasis samples were analysed. In fact, for theranostic purposes and in order to include patients with metastatic tumours in early trials to give matched targeted therapy, molecular analyses on metastasis seem more relevant. However, it could be interesting to perform the same study on primary tumours as they might have a higher variability of clones than in the metastasis, the latest originating from one major clone under treatment selection. Indeed, two samples (CNB and FNA) taken from the primitive tumour could harbour a higher heterogeneity and show more molecular discordances compared to the metastasis [37, 38].

Four patients out of 61 did not harbour any relevant validated molecular alteration (patients 1, 23, 41 and 45). For these cases, sampling procedures were successful, except for patient 45 for which the biopsied lesion appeared to be a nontumoral lymph node rather than a metastasis (which was lately confirmed by PET scan). Histologies were validated by the pathologists, and no anomaly was reported during NGS process. The absence of validated molecular alterations in these samples could therefore either be explained by the absence of alterations at the DNA level in these tumours, or by a lack of coverage of the altered genes in the targeted panel used.

Two patients had targetable alterations detected only in FNA: patient 19 and patient 27. Patient 19 had a homozygous deletion of *CDKN2A* only detected in the FNA, while a heterozygous deletion of *CDKN2A* was found in the CNB. This result still led to orientation to a treatment by CDK4‐6 inhibitor (Palbociclib) in the frame of SHIVA‐02, but unfortunately, the patient died one month later. Patient 27 harboured an homozygous deletion of *PTEN* in FNA sample only but not in the CNB. This result did not affect the decision taken within the SHIVA02 molecular board (which is only based on alterations found in CNB), as *PTEN* inactivation is not part of the treatment algorithm. Yet, this new result led to a discussion in the local Molecular Tumour Board (MTB) for eventual treatment by mTOR inhibitor [39].

These discordances between molecular results from CNB and FNA could be explained by intratumoral heterogeneity [37, 38, 40], as CNB and FNA were taken from spatially separated points within the tumour (see Tumour sample collection and processing).

Among validated alterations across all the samples, *TP53, PIK3CA, KRAS, ESR1* and *CDKN2A/B* were the most frequently altered genes, which is in accordance with the tumour types screened, as the majority were metastatic breast, colon and pancreas cancers [41, 42]. Regarding targetable alterations found among validated data, the same repartition of alterations across the different cellular pathways was observed between CNB and FNA samples. The most affected signalling pathways were the MAPK and the PTEN/PI3K/AKT/mTOR pathways, which are related to the most frequently altered genes found among the samples, and are in accordance with the genomic characteristics of the tumour types analysed (i.e., mainly breast, pancreas and colon cancers) [43, 44]. These observations reflect once again the relevance of the data validation step and confirm the reliability of FNA samples for detection of relevant and actionable molecular alterations in patients suffering from advanced cancer.

## 5 Conclusions

Our results show that: (a) both CNB and FNA samples are suitable for molecular analysis, with the same DNA and NGS qualities; (b) relevant validated molecular alterations are equally detected in both CNB and FNA samples from the same patient, with a higher consistency after than before validation of data; and (c) actionable targets were identified in both CNB and FNA. We demonstrated the feasibility of real‐time molecular screening of biomarkers from FNA samples and incorporation into clinical workflows to orientate diagnosis or therapeutic decisions. Finally, our study also emphasizes the critical role of data analysis and validation of relevant molecular alterations in order to have reliable, comparable data between different sample types.

With the increasing need to identify tumour‐specific molecular alterations to improve diagnosis and treatment algorithms for personalized therapy, FNA samples can be of critical utility, especially for patients with nonbiopsiable lesions or advanced diseases where small specimen is the only material available, or, when performed in parallel to CNB, molecular profiling on FNA could allow to save tissue material for other purposes. This could open the doors of NGS analyses and new therapeutic options to more patients.

## Author contributions

IB, VS, JM‐P, MK and CLT conceived and designed the study. JM‐P, EG, PG, VS and MK developed the methodology. JM‐P, MK, SA, CF, OM, EG, PG, PR, EB, DB, FR, SH, M‐PS, PT, AM, DL, MF, AV‐S and CG involved in data acquisition. JM‐P, EG, PG and CD analysed and interpreted the data. CD, JM‐P, MK, CG, IB and CLT wrote, reviewed and revised the manuscript. IB, CLT, AV‐S, JM‐P, CD, MK, CG, EG, PG, CC, SA, CF, OM and CL gave administrative, technical or material support (i.e., reporting or organizing data, constructing databases). IB, VS, CLT and MK supervised the study.

## Conflict of interest

CLT has participated in advisory boards from MSD, BMS, Merck Serono, GSK, Amgen, Novartis, Roche, Rakuten, Seattle Genetics, Nanobiotix and Astra Zeneca.

## Supporting information


**Fig. S1.** Representation of sample acquisition in cancer patients. Concomitant FNA and CNB are performed during the same procedure. A 22 Gauge needle is inserted into the guide needle to perform the first sampling (1) which is the FNA. Then, an 18 or 16 Gauge cutting needle is inserted into the guide needle to perform the CNB. The second sampling corresponding to CNB is performed close from the first FNA sampling, either by going deeper into the tissue with the needle (2) or changing the needle's angle (2′).

**Fig. S2.** Percentages of tumour cells in CNB and FNA samples determined by pathologists. Data are presented as mean ± SEM. Mann–Whitney test was used to compare the group of CNB samples versus FNA samples.

**Fig. S3.** Repartition of DNA concentration ranges in CNB and FNA samples analysed. Data are presented as mean ± SD. Kruskal–Wallis test followed by Dunn's test was used to compare CNB and FNA samples DNA concentrations within the 3 groups (<50 ng/µl; 50‐100 ng/µl and >100 ng/µl) and no significance was found.

**Fig. S4.** Number of pathogenic variants detected in CNB and FNA per patient.

**Table S1.** Cancer type, tumour cell percentage, and DNA and NGS libraries quality control metrics of CNB and FNA samples.
**Table S2.** Description of validated molecular alterations in CNB and FNA samples of 61 patients from the SHIVA02 trial.

## Data Availability

The data sets used and/or analysed during the current study are available from the corresponding author on reasonable request.
