# Supplementary figures and images for: Fine‐needle aspiration as an alternative to core needle biopsy for tumour molecular profiling in precision oncology: prospective comparative study of next‐generation sequencing in cancer patients included in the SHIVA02 trial
Source: Mol Oncol. 2020 Sep 15;15(1):104–15. doi: 10.1002/1878-0261.12776 (PMC7782085; doi:10.1002/1878-0261.12776)

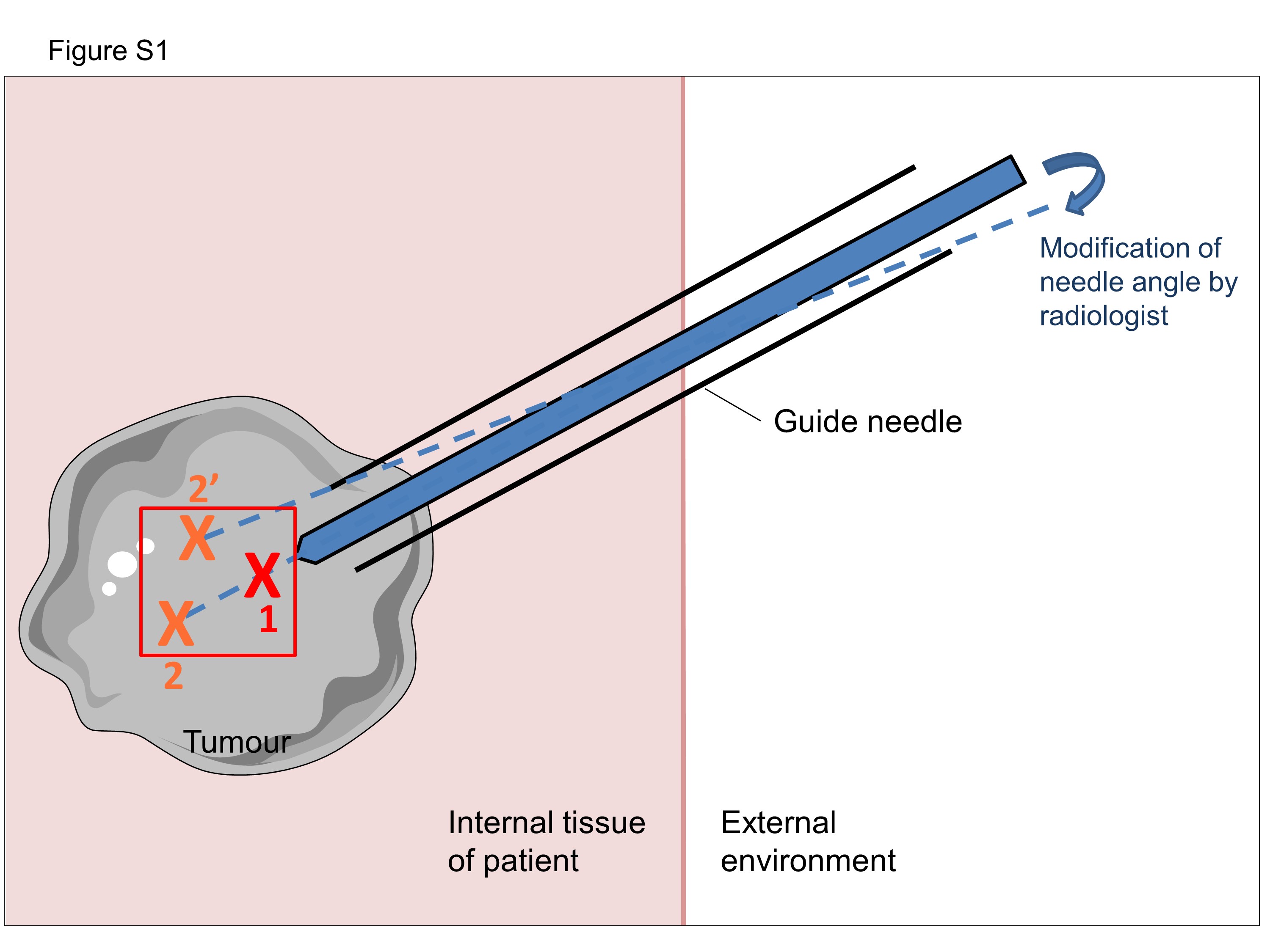

Supplement: Supplementary file 1 — Fig. S1. Representation of sample acquisition in cancer patients. Concomitant FNA and CNB are performed during the same procedure. A 22 Gauge needle is inserted into the guide needle to perform the first sampling (1) which is the FNA. Then, an 18 or 16 Gauge cutting needle is inserted into the guide needle to perform the CNB. The second sampling corresponding to CNB is performed close from the first FNA sampling, either by going deeper into the tissue with the needle (2) or changing the needle's angle (2′). [file MOL2-15-104-s001.jpg]

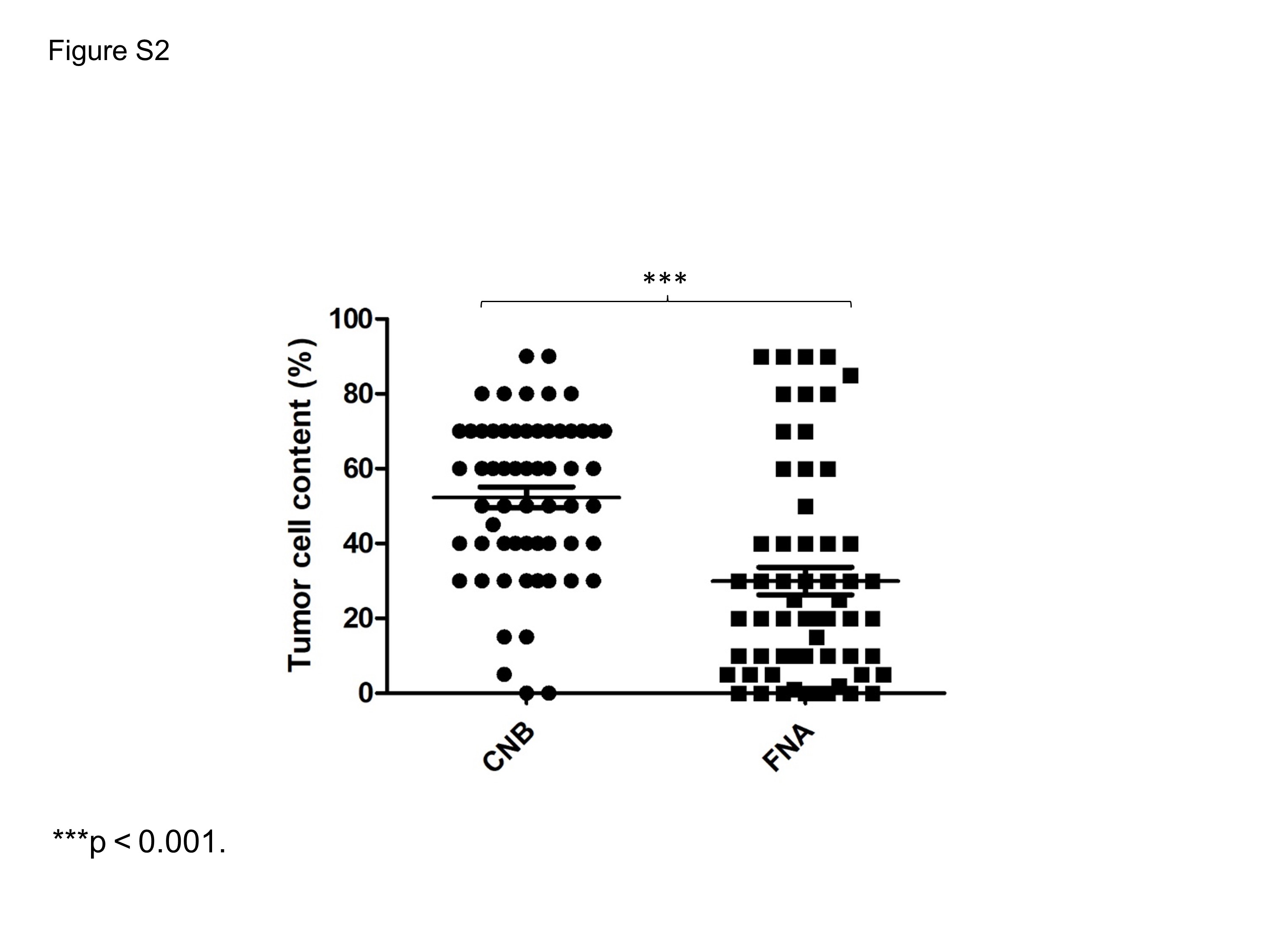

Supplement: Supplementary file 2 — Fig. S2. Percentages of tumour cells in CNB and FNA samples determined by pathologists. Data are presented as mean ± SEM. Mann–Whitney test was used to compare the group of CNB samples versus FNA samples. [file MOL2-15-104-s002.jpg]

Figure S3

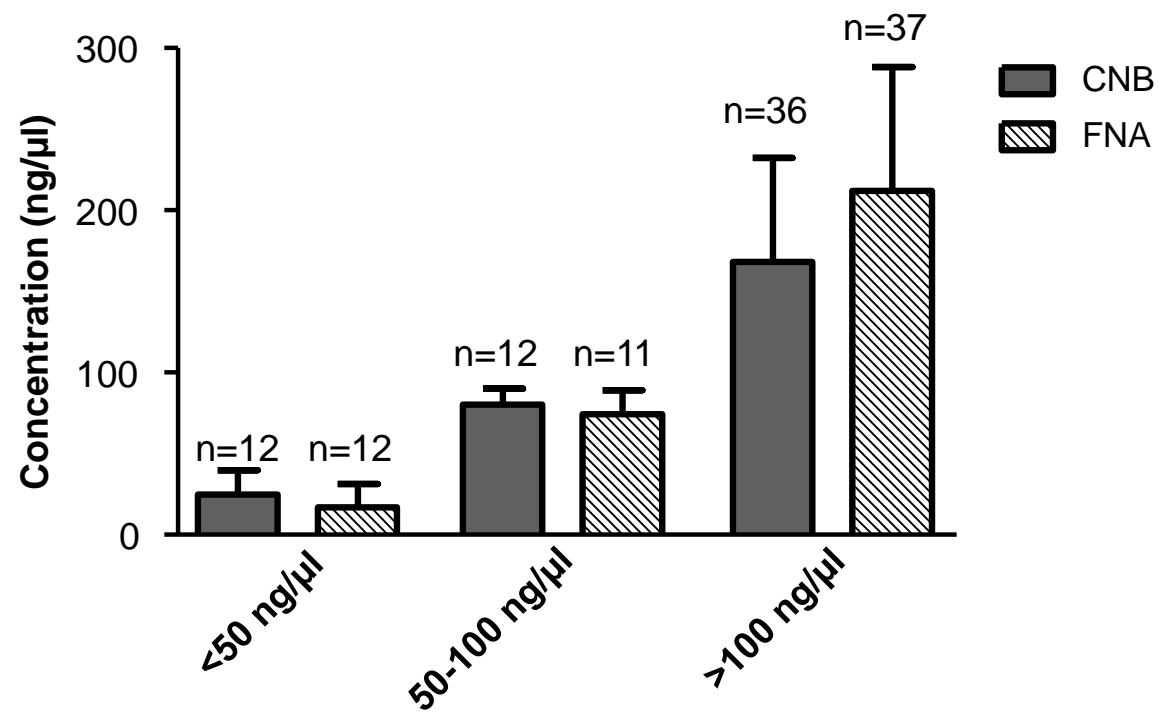

Supplement: Supplementary file 3 — Fig. S3. Repartition of DNA concentration ranges in CNB and FNA samples analysed. Data are presented as mean ± SD. Kruskal–Wallis test followed by Dunn's test was used to compare CNB and FNA samples DNA concentrations within the 3 groups (<50 ng/µl; 50‐100 ng/µl and >100 ng/µl) and no significance was found. [file MOL2-15-104-s003.pdf]

Figure S4

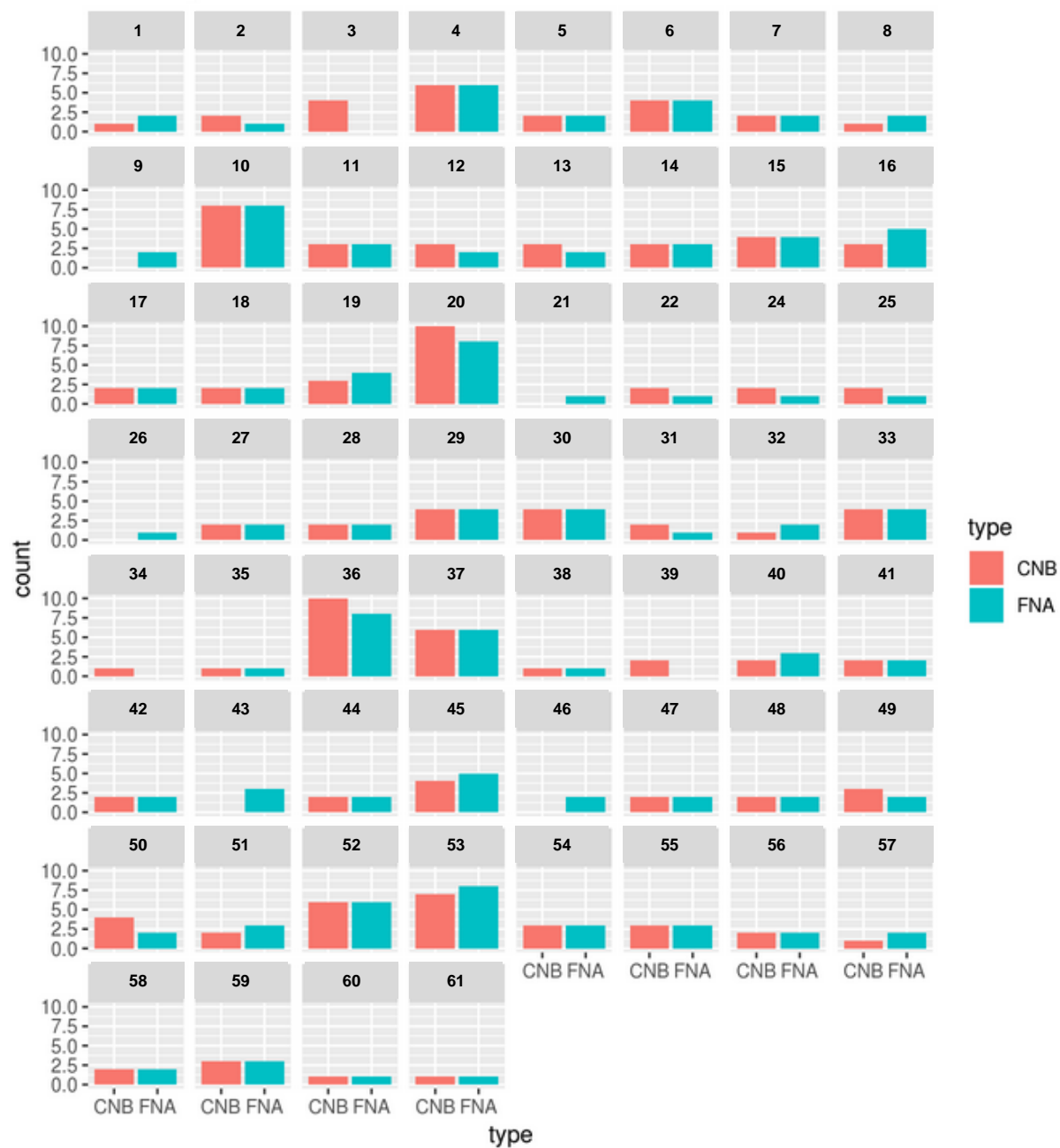

Supplement: Supplementary file 4 — Fig. S4. Number of pathogenic variants detected in CNB and FNA per patient. [file MOL2-15-104-s004.pdf]
