# Supplementary material for: Fine‐needle aspiration as an alternative to core needle biopsy for tumour molecular profiling in precision oncology: prospective comparative study of next‐generation sequencing in cancer patients included in the SHIVA02 trial
Source: Mol Oncol. 2020 Sep 15;15(1):104–15. doi: 10.1002/1878-0261.12776 (PMC7782085; doi:10.1002/1878-0261.12776)
Supplement: Supplementary file 5 — Table S1. Cancer type, tumour cell percentage, and DNA and NGS libraries quality control metrics of CNB and FNA samples. Table S2. Description of validated molecular alterations in CNB and FNA samples of 61 patients from the SHIVA02 trial. [file MOL2-15-104-s005.docx]

**Table S1: Cancer type, tumour cell percentage, and DNA and NGS libraries quality control metrics of CNB and FNA samples.**

^1^ Measured using Qubit™; ^2^ Presence of a smear in gel indicating a small degradation of DNA. CNB = Core Needle Biopsy; FNA = Fine Needle Aspiration; NGS = Next-Generation Sequencing. Conc = Concentration.

Mann-Whitney test was used to compare CNB and FNA samples regarding : DNA concentrations, DNA ratios (both 260/280 and 260/230), reads number, the percentage of mapped reads, the percentage of mapped reads on target and the percentage of depth≥100. No statistical difference was found.

| **Patient number** | **Primary tumor localiza-**  **tion** | **Primary tumor histological subtype** | **Biopsy site** | **Tumor cell content** | | **DNA characteristics** | | | | | | | | **NGS quality characteristics** | | | | | | | |
| --- | --- | --- | --- | --- | --- | --- | --- | --- | --- | --- | --- | --- | --- | --- | --- | --- | --- | --- | --- | --- | --- |
|  |  |  |  | **CNB** | **FNA** | **CNB** | | | | **FNA** | | | | **CNB** | | | | **FNA** | | | |
|  |  |  |  |  |  | **Conc. Qubit (ng/µl) ^1^** | **Ratio 260/280** | **Ratio 260/230** | **Quality on gel** | **Conc. Qubit (ng/µl) ^1^** | **Ratio 260/280** | **Ratio 260/230** | **Quality on gel** | **Reads Number** | **Mapped Reads**  **(%)** | **Mapped Reads on Target**  **(%)** | **Depth ≥100(%)** | **Reads Number** | **Mapped Reads**  **(%)** | **Mapped Reads on Target**  **(%)** | **Depth ≥100(%)** |
| 1 | parotid | mucoepidermoid carcinoma | liver | 50% | 20% | 121,0 | 1.85 | 1.64 | OK | 278,0 | 1.87 | 2.09 | OK | 12 140 442 | 94.3 | 91.09 | 79.49 | 9 602 348 | 92.94 | 89.76 | 76.53 |
| 2 | ovary | serous adenocarcinoma | liver | 80% | 20% | 216,0 | 1.87 | 2.29 | OK | 414,0 | 1.85 | 2.21 | OK + smear^2^ | 2 844 796 | 98.63 | 92.1 | 89.94 | 4 502 348 | 98.37 | 92.33 | 91.72 |
| 3 | breast | invasive carcinoma NST | lymph node | 0% | 90% | 15.7 | 1.68 | 0.5 | OK | ND | 1.15 | 0.12 | ND | 9 877 082 | 58.89 | 54.42 | 96.06 | 6 449 418 | 71.21 | 65.1 | 90.27 |
| 4 | colon | adenocarcinoma | liver | 70% | 0% | 88.9 | 1.87 | 2.28 | OK | 139,0 | 1.86 | 2.52 | OK | 10 496 406 | 88.78 | 81.22 | 96.71 | 13 078 764 | 90.8 | 81.76 | 97.76 |
| 5 | pancreas | ductal adenocarcinoma | liver | 60% | 1% | 112,0 | 1.85 | 1.8 | OK | 132,0 | 1.85 | 2.43 | OK | 19 095 070 | 97.04 | 89.72 | 97.96 | 13 380 528 | 94.85 | 85.91 | 97.76 |
| 6 | breast | invasive carcinoma NST | sub-cutaneous | 70% | 80% | 145,0 | 1.95 | 2.26 | OK | 81,0 | 1.85 | 2.76 | OK | 20 360 756 | 97.33 | 90.11 | 98.16 | 20 414 668 | 95.35 | 88.4 | 98.03 |
| 7 | prostate | adenocarcinoma | liver | 40% | 10% | 253,0 | 1.92 | 2.35 | OK | 78.2 | 1.85 | 1.21 | OK | 21 051 940 | 94.54 | 87.15 | 97.83 | 11 173 110 | 92.63 | 85.76 | 96.78 |
| 8 | breast | invasive carcinoma NST | liver | 70% | 15% | 361,0 | 1.89 | 1.99 | OK | 76.1 | 1.77 | 2.01 | OK | 15 902 302 | 98.48 | 89.66 | 97.9 | 12 093 516 | 94.81 | 86.23 | 97.44 |
| 9 | pancreas | ductal adenocarcinoma | liver | 60% | 30% | 102,0 | 1.88 | 1.51 | OK | 139,0 | 1.82 | 1.55 | OK | 5 363 278 | 6.68 | 4.97 | 27.09 | 11 047 166 | 12.6 | 9.57 | 83.83 |
| 10 | stomach | adenocarcinoma | liver | 30% | 40% | 76.7 | 1.89 | 1.82 | OK | 209,0 | 1.86 | 2.24 | OK | 12 823 906 | 75.72 | 69.06 | 97.7 | 12 087 916 | 77.89 | 67.53 | 97.83 |
| 11 | breast | invasive carcinoma NST | lung | 60% | 85% | 155,0 | 1.95 | 2.24 | OK | 114,0 | 1.9 | 2.05 | OK | 11 810 696 | 22.47 | 20.04 | 92.9 | 11 794 020 | 17.69 | 15.47 | 90.4 |
| 12 | breast | lobular and invasive carcinoma NST | liver | 60% | 0% | 157,0 | 1.85 | 2.04 | OK | 16.5 | 1.69 | 1.14 | OK | 9 994 124 | 31.93 | 24.53 | 93.1 | 8 814 722 | 93.94 | 85.27 | 96.32 |
| 13 | colon | adenocarcinoma | liver | 60% | 40% | 189,0 | 1.82 | 2.15 | OK | 54.9 | 1.84 | 1.65 | OK | 6 027 806 | 87.39 | 81.35 | 95.6 | 5 944 302 | 67.39 | 62.2 | 90.34 |
| 14 | lung | adenocarcinoma | lymph node | 40% | 20% | 19.5 | 1.83 | 1.93 | OK | 3.21 | 1.69 | 1.68 | OK | 6 097 258 | 87.73 | 78.7 | 95.6 | 10 916 994 | 90,00 | 81.83 | 97.3 |
| 15 | colon | adenocarcinoma | peritoneum | 70% | 10% | 68.8 | 1.85 | 1.82 | OK | 5.26 | 1.66 | 2.36 | OK | 10 191 992 | 97.27 | 90.84 | 95.33 | 11 316 878 | 91.31 | 84.07 | 97.57 |
| 16 | pancreas | ductal adenocarcinoma | lung | 40% | 30% | 64.9 | 1.82 | 1.46 | OK | 98.3 | 1.64 | 1.81 | OK | 10 821 824 | 96.39 | 88.84 | 96.98 | 6 662 382 | 83.12 | 77,00 | 95.4 |
| 17 | uterus | endometrial serous adenocarcinoma | liver | 90% | 25% | 120,0 | 1.9 | 2.13 | OK | 166,0 | 1.86 | 2.2 | OK | 13 177 536 | 31.41 | 27.09 | 94.02 | 15 869 244 | 14.15 | 11.8 | 89.74 |
| 18 | breast | invasive carcinoma NST | liver | 70% | 20% | 154,0 | 1.87 | 2.02 | OK | 110,0 | 1.88 | 2.26 | OK | 16 752 748 | 89.68 | 76.84 | 98.29 | 2 042 040 | 85.1 | 77.9 | 89.02 |
| 19 | esophagus | squamous cell carcinoma | liver | 70% | 25% | 33.4 | 1.87 | 2.05 | OK | 353,0 | 1.87 | 2.17 | OK + smear^2^ | 9 214 532 | 52.16 | 42.22 | 95.33 | 1 227 758 | 81.08 | 74.53 | 80.34 |
| 20 | colon | squamous cell carcinoma | peritoneum | 0% | 0% | 0,0 | 1.57 | 0.49 | not visible | 30.5 | 1.84 | 2.83 | OK | 12 987 478 | 82.57 | 73.42 | 97.83 | 10 832 146 | 95.7 | 88.35 | 97.96 |
| 21 | stomach | liposarcoma dedifferenciated | sub-cutaneous | 90% | 90% | 265,0 | 1.89 | 2.4 | OK | 296,0 | 1.91 | 2.32 | OK | 12 748 890 | 94.88 | 87.77 | 97.63 | 13 924 718 | 95.07 | 85.7 | 97.76 |
| 22 | breast | invasive carcinoma NST | liver | 45% | 2% | 165,0 | 1.86 | 2.12 | OK | 173,0 | 1.87 | 2.64 | OK | 12 570 658 | 94.11 | 86.04 | 97.76 | 13 074 202 | 94.39 | 86.11 | 97.24 |
| 23 | cervix | neuroendocrine carcinoma (high grade) | liver | 70% | 60% | 29.6 | 1.73 | 2.21 | OK | 177,0 | 1.87 | 2.29 | OK | 22 164 826 | 85,00 | 78.65 | 98.03 | 5 300 404 | 95.71 | 87.46 | 95.73 |
| 24 | pancreas | ductal adenocarcinoma | sub-cutaneous | 60% | 30% | 203,0 | 1.88 | 2.16 | ND | 228,0 | 1.87 | 1.85 | ND | 11 178 332 | 87.53 | 79.89 | 97.17 | 4 551 410 | 95.93 | 86.95 | 94.74 |
| 25 | breast | neuroendocrine carcinoma | liver | 70% | 70% | 118,0 | 1.85 | 2.44 | OK | 229,0 | 1.82 | 1.94 | OK | 9 089 680 | 63.39 | 58.65 | 94.81 | 14 068 502 | 74.35 | 69.35 | 96.45 |
| 26 | stomach | adenocarcinoma | liver | 40% | 30% | 230,0 | 1.88 | 2.25 | OK | 292,0 | 1.89 | 2.21 | OK | 12 831 342 | 96.41 | 88.77 | 98.16 | 11 694 506 | 94.76 | 86.7 | 97.3 |
| 27 | colon | squamous cell carcinoma | peritoneum | 80% | 70% | 14.5 | 1.88 | 1.42 | OK | 3.89 | 1.76 | 1.15 | OK | 12 199 378 | 94.53 | 87.31 | 97.9 | 4 630 402 | 91.31 | 83.69 | 94.61 |
| 28 | cervix | squamous cell carcinoma | liver | 40% | 30% | 72.5 | 1.89 | 1.54 | OK | 222,0 | 1.87 | 1.8 | OK | 9 859 978 | 80.35 | 74.43 | 97.63 | 11 465 904 | 55.9 | 51.51 | 96.78 |
| 29 | breast | invasive carcinoma NST | liver | 70% | 5% | 88.1 | 1.91 | 2.06 | OK | 322,0 | 1.88 | 2.3 | OK | 12 692 120 | 96.83 | 89.34 | 98.22 | 15 618 222 | 95.95 | 87.75 | 98.22 |
| 30 | pancreas | ductal adenocarcinoma | sub-cutaneous | 60% | 60% | 265,0 | 1.9 | 2.19 | OK | 218,0 | 1.89 | 2.3 | OK | 6 687 814 | 96.02 | 87.51 | 96.32 | 8 313 688 | 97.02 | 88.73 | 96.65 |
| 31 | uterus | epithelioid sarcoma | liver | 70% | 30% | 35,0 | 1.89 | 1.67 | OK | 332,0 | 1.87 | 2.21 | OK | 14 875 998 | 96.57 | 89.09 | 97.44 | 13 799 790 | 92.34 | 84.28 | 97.7 |
| 32 | breast | invasive carcinoma NST | sub-cutaneous | 30% | 10% | 73.4 | 1.8 | 1.96 | OK | 152,0 | 1.91 | 2.16 | OK | 18 100 674 | 82.59 | 75.73 | 97.5 | 21 584 380 | 42.02 | 38.43 | 96.45 |
| 33 | colon | adenocarcinoma | liver | 50% | 5% | 237,0 | 1.89 | 2.25 | OK | 7.5 | 1.72 | 1.39 | OK | 17 255 400 | 81.88 | 76.78 | 96.65 | 13 683 158 | 95.42 | 87.44 | 97.63 |
| 34 | bladder | adenocarcinoma | liver | 30% | 5% | 96.6 | 1.86 | 2.09 | OK | 62.7 | 1.83 | 2.3 | OK | 13 232 698 | 96.9 | 89.39 | 97.63 | 19 612 096 | 85.63 | 78.58 | 97.44 |
| 35 | breast | lobular carcinoma | liver | 40% | 20% | 24.9 | 1.83 | 2.77 | OK | 12.2 | 1.71 | 3.6 | OK | 7 715 748 | 54.78 | 47.87 | 94.81 | 12 255 358 | 95,00 | 84.53 | 96.91 |
| 36 | colon | adenocarcinoma | liver | 50% | 10% | 144,0 | 1.92 | 1.88 | OK | 122,0 | 1.89 | 2.15 | OK | 11 226 016 | 90.27 | 79.46 | 97.3 | 13 026 776 | 94.94 | 85.41 | 97.24 |
| 37 | breast | invasive carcinoma NST | lung | 70% | 10% | 187,0 | 1.72 | 2.1 | OK | 44,0 | 1.77 | 2.06 | OK | 14 236 526 | 86.67 | 76.92 | 97.63 | 14 230 404 | 86.72 | 77.74 | 97.37 |
| 38 | breast | invasive carcinoma NST | sub-cutaneous | 80% | 40% | 143,0 | 1.88 | 2.16 | OK | 28.7 | 1.83 | 1.44 | OK | 13 380 244 | 86.78 | 78.22 | 97.24 | 13 205 496 | 86.24 | 77.46 | 96.98 |
| 39 | breast | invasive carcinoma NST | lung | NE | 70% | 0,0 | 1.48 | 0.57 | OK | 197,0 | 1.87 | 2,00 | OK | 10 375 416 | 84.63 | 73.3 | 96.84 | 16 578 908 | 81.1 | 73.95 | 97.76 |
| 40 | breast | invasive carcinoma NST | liver | 50% | 10% | 123,0 | 1.86 | 2.08 | OK | 186,0 | 1.87 | 2.19 | OK | 12 350 380 | 24.89 | 22.59 | 94.15 | 11 744 038 | 19.31 | 17.66 | 91.12 |
| 41 | cervix | adenocarcinoma | lymph node | 30% | 0% | 86.5 | 1.86 | 2.21 | OK | 2.87 | 3.05 | 0.72 | OK | 14 340 886 | 20.06 | 18.35 | 93.16 | 13 381 512 | 40.49 | 36.96 | 96.06 |
| 42 | muscle | leiomyosarcoma | liver | 70% | 0% | 111,0 | 1.91 | 2.21 | OK | 72.4 | 1.9 | 1.51 | OK | 15 235 806 | 94.3 | 85.88 | 98.29 | 10 975 380 | 68.77 | 61.54 | 96.71 |
| 43 | lung | spindle cell carcinoma | peritoneum | 40% | 40% | 74.1 | 1.82 | 2.11 | OK | 53.3 | 1.81 | 2.03 | OK | 8 144 454 | 1.46 | 1,00 | 3.88 | 12 862 900 | 80.24 | 73.53 | 97.04 |
| 44 | breast | invasive carcinoma NST | lymph node | 80% | 80% | 130,0 | 1.93 | 1.87 | OK | 245,0 | 1.83 | 1.73 | OK | 11 442 036 | 87.52 | 79.23 | 97.5 | 15 846 450 | 84.93 | 78.65 | 97.76 |
| 45 | bladder | urothelial carcinoma (high grade) | breast | 80% | 0% | 244,0 | 1.93 | 2.27 | OK | 9,0 | 1.79 | 1.92 | OK | 14 313 858 | 95.14 | 86.63 | 98.03 | 11 154 704 | 86.61 | 79.28 | 96.19 |
| 46 | pancreas | ductal adenocarcinoma | liver | 30% | 0% | 131,0 | 1.9 | 2,00 | OK + smear^2^ | 63,0 | 1.81 | 1.45 | OK | 9 768 676 | 96.03 | 84.53 | 97.44 | 12 638 082 | 91.33 | 82.82 | 97.76 |
| 47 | cervix | adenocarcinoma of the uterine cervix | lymph node | 70% | 90% | 89.3 | 1.832 | 1.81 | OK + smear^2^ | 182,0 | 7.35 | 2.27 | OK | 2 948 176 | 98.27 | 89.39 | 93.36 | 2 948 176 | 98.27 | 89.39 | 93.36 |
| 48 | breast | metaplastic carcinoma | lymph node | 30% | 90% | 101,0 | 1.93 | 2.17 | OK | 187,0 | 1.65 | 0.96 | OK | 6 767 060 | 97.83 | 87.83 | 96.19 | 16 920 820 | 96.44 | 88.18 | 97.76 |
| 49 | breast | invasive carcinoma NST | liver | 60% | 60% | 289,0 | 1.87 | 2.26 | OK | 360,0 | 1.87 | 2.31 | OK | 8 450 670 | 97.7 | 90.81 | 96.58 | 12 598 084 | 88.94 | 81.91 | 97.63 |
| 50 | breast | invasive carcinoma NST | liver | 40% | 5% | 129,0 | 1.87 | 2.04 | OK | 152,0 | 1.87 | 2.19 | OK | 11 112 082 | 97.35 | 90,00 | 97.11 | 11 287 396 | 96.45 | 89.02 | 97.44 |
| 51 | breast | invasive carcinoma NST and lobular | liver | 60% | 20% | 104,0 | 1.85 | 1.63 | OK | 224,0 | 1.87 | 2.26 | ND | 11 309 898 | 95.79 | 89.72 | 97.37 | 11 360 294 | 97.14 | 90.97 | 97.5 |
| 52 | breast | invasive carcinoma NST | liver | 30% | 10% | 45,0 | 1.81 | 2.14 | OK | 150,0 | 1.86 | 2.19 | OK | 10 409 384 | 94.48 | 86.35 | 97.44 | 11 561 410 | 94.51 | 86.74 | 97.57 |
| 53 | breast | lobular carcinoma | liver | 15% | 5% | 41,0 | 1.74 | 0.96 | ND | 237,0 | 1.87 | 2.19 | OK | 11 702 344 | 88.37 | 81.93 | 97.5 | 11 193 408 | 94.76 | 87.49 | 97.37 |
| 54 | colon | mucinous adenocarcinoma | peritoneum | 15% | 20% | 158,0 | 1.9 | 1.92 | OK | 90,0 | 1.84 | 0.9 | OK | 6 233 978 | 96.11 | 89.01 | 96.45 | 7 028 490 | 96.51 | 89.27 | 96.25 |
| 55 | breast | lobular carcinoma | liver | 50% | 0% | 179,0 | 1.9 | 2.15 | OK | 256,0 | 2.994 | 1.87 | OK | 7 878 912 | 97.01 | 88.97 | 96.84 | 7 878 912 | 97.01 | 88.97 | 96.84 |
| 56 | stomach | ductal adenocarcinoma | liver | 5% | 40% | ND | ND | ND | ND | 143,0 | 1.87 | 2.29 | OK | 10 621 192 | 94.45 | 85.7 | 97.63 | 6 235 842 | 95.04 | 84.91 | 96.38 |
| 57 | pancreas | ductal adenocarcinoma | liver | 30% | 80% | 38.8 | 1.81 | 1.76 | OK | 116,0 | 1.85 | 2.22 | OK | 648 474 | 93.81 | 84.65 | 61.47 | 9 655 338 | 96.45 | 88.76 | 97.11 |
| 58 | breast | invasive carcinoma NST | lymph node | 60% | 30% | 101,0 | 1.82 | 2.08 | OK | 37.4 | 1.63 | 1.63 | OK | 9 547 026 | 94.5 | 87.55 | 96.38 | 9 547 026 | 94.5 | 87.55 | 96.91 |
| 59 | colon | adenocarcinoma | liver | 40% | 10% | 103,0 | 1.86 | 1.83 | OK + smear^2^ | 88.1 | 1.88 | 1.68 | OK | 8 057 012 | 91.86 | 81.43 | 96.65 | 11 506 434 | 94.07 | 86.89 | 97.5 |
| 60 | uterus | carcinosarcoma | peritoneum | 50% | 20% | 120,0 | 1.87 | 2.27 | OK | 184,0 | 1.9 | 2.19 | OK | 12 857 524 | 95.39 | 86.35 | 97.83 | 13 937 842 | 95.05 | 85.65 | 97.9 |
| 61 | bone | ewing sarcoma | lung | 70% | 50% | 84.3 | 1.86 | 2.22 | OK | 210,0 | 1.89 | 2.33 | OK | 13 441 114 | 96.73 | 87.66 | 97.96 | 13 536 494 | 97.12 | 88.29 | 97.57 |

**Table S2: Description of validated molecular alterations in CNB and FNA samples of 61 patients from the SHIVA02 trial.**

| **Patient number** | **CNB** | | | | | | | | **FNA** | | | | | | | | | | **Conclusion between CNB and FNA** |
| --- | --- | --- | --- | --- | --- | --- | --- | --- | --- | --- | --- | --- | --- | --- | --- | --- | --- | --- | --- |
|  | **Mutations** | | | | | | **CNV** | | **Mutations** | | | | | | | **CNV** | | |  |
|  | **Gene** | **Nucleotide variation** | **Protein variation** | **X** | **Allelic ratio** | **Conclusion mutation** | **Log2 ratio** | **Conclusion CNV** | **Gene** | **Nucleotide variation** | **Protein variation** | **X** | **Allelic ratio** | **Conclusion mutation** | **Log2 ratio** | | **Conclusion CNV** |  | |
| 1 | NA | NA | NA | NA | NA | NA | NA | NA | NA | NA | NA | NA | NA | NA | NA | | NA | Concordant | |
| 2 | TP53 | c.475G>C | p.(Ala159Pro) | 860 | 45,4% | Homozygous | 0,513 | Homozygous deletion | *TP53* | c.475G>C | p.(Ala159Pro) | 1764 | 30,6% | Heterozygous | 0,668 | | Normal | Discordant | |
| 2 | INPP4B | NA | NA | NA | NA | NA | -2,601 | Homozygous deletion | *INPP4B* | NA | NA | NA | NA | NA | -0,655 | | Homozygous deletion | Discordant | |
| 2 | PIK3R1 | NA | NA | NA | NA | NA | -1,433 | Homozygous deletion | *PIK3R1* | NA | NA | NA | NA | NA | -1,006 | | Homozygous deletion | Discordant | |
| 3 | ERBB2 | NA | NA | NA | NA | NA | 3,47 | Amplification | *ERBB2* | NA | NA | NA | NA | NA | 5,022 | | Amplification | Concordant | |
| 3 | MDM2 | NA | NA | NA | NA | NA | 2,866 | Amplification | *MDM2* | NA | NA | NA | NA | NA | 4,668 | | Amplification | Concordant | |
| 3 | FGFR1 | NA | NA | NA | NA | NA | 1,275 | Gain | *FGFR1* | NA | NA | NA | NA | NA | 2,257 | | Amplification | Discordant | |
| 4 | TP53 | c.859G>T | p.(Glu287*) | 1530 | 77,1% | Homozygous | 0,115 | Normal | *TP53* | c.859G>T | p.(Glu287*) | 2126 | 72% | Homozygous | 0,21 | | Normal | Concordant | |
| 4 | MAP2K1 | c.170A>C | p.(Lys57Thr) | 6585 | 29,3% | Heterozygous | 0,115 | Normal | *MAP2K1* | c.170A>C | p.(Lys57Thr) | 8004 | 21% | Heterozygous | 0,211 | | Normal | Concordant | |
| 4 | CDKN2A/2B | NA | NA | NA | NA | NA | -0,73 | Homozygous deletion | *CDKN2A/2B* | NA | NA | NA | NA | NA | -1,27 | | Homozygous deletion | Concordant | |
| 5 | TP53 | c.818G>A | p.(Arg273His) | 4752 | 36,1% | Heterozygous | -0,231 | Normal | *TP53* | c.818G>A | p.(Arg273His) | 2043 | 11% | Heterozygous | -0,077 | | Normal | Concordant | |
| 5 | KRAS | c.35G>A | p.(Gly12Asp) | 3728 | 59,7% | Heterozygous | 0,184 | Gain | *KRAS* | c.35G>A | p.(Gly12Asp) | 1236 | 24% | Heterozygous | 0,026 | | Normal | Concordant | |
| 5 | PIK3CA | c.1624G>A | p.(Glu542Lys) | 8588 | 0,0% | Absent | 0,093 | Gain | *PIK3CA* | c.1624G>A | p.(Glu542Lys) | 4177 | 3% | Heterozygous | 0,056 | | Normal | Discordant | |
| 6 | ESR1 | c.1138G>C | p.(Glu380Gln) | 2300 | 30,0% | Heterozygous | -0,695 | Homozygous deletion | *ESR1* | c.1138G>C | p.(Glu380Gln) | 1839 | 39,7% | Heterozygous | -0,739 | | Homozygous deletion | Concordant | |
| 6 | ESR1 | c.1610A>G | p.(Tyr537Cys) | 400 | 36,5% | Heterozygous | -0,695 | Homozygous deletion | *ESR1* | c.1610A>G | p.(Tyr537Cys) | 442 | 51,4% | Heterozygous | -0,739 | | Homozygous deletion | Concordant | |
| 6 | FGFR1 | NA | NA | NA | NA | NA | 1,341 | Amplification | *FGFR1* | NA | NA | NA | NA | NA | 1,373 | | Amplification | Concordant | |
| 6 | PTEN | c.389G>A | p.(Arg130Gln) | 3825 | 47,7% | Heterozygous | -0,228 | Normal | *PTEN* | c.389G>A | p.(Arg130Gln) | 3739 | 64,5% | Homozygous | -0,223 | | Normal | Discordant | |
| 7 | AR | NA | NA | NA | NA | NA | 1,39 | Amplification | *AR* | NA | NA | NA | NA | NA | 1,247 | | Amplification | Concordant | |
| 7 | TP53 | c.376-1G>T | p.? | 1328 | 76,7% | Homozygous | -0,531 | Homozygous deletion | *TP53* | c.376-1G>T | p.? | 417 | 91,1% | Homozygous | -0,698 | | Homozygous deletion | Concordant | |
| 8 | ESR1 | c.1607T>A | p.(Leu536His) | 438 | 31,5% | Homozygous | -0,366 | Homozygous deletion | *ESR1* | c.1607T>A | p.(Leu536His) | 173 | 29,5% | Homozygous | -0,265 | | Homozygous deletion | Concordant | |
| 9 | KRAS | c.35G>A | p.(Gly12Asp) | 37 | 29,7% | Heterozygous | 0,007 | Normal | *KRAS* | c.35G>A | p.(Gly12Asp) | 174 | 2,0% | WT | 0,017 | | Normal | Discordant | |
| 10 | CCNE1 | NA | NA | NA | NA | NA | 2,507 | Amplification | *CCNE1* | NA | NA | NA | NA | NA | 1,503 | | Amplification | Concordant | |
| 10 | PIK3CA | c.1634A>C | p.(Glu545Ala) | 3509 | 21,4% | Heterozygous | 0,38 | Normal | *PIK3CA* | c.1634A>C | p.(Glu545Ala) | 3491 | 12,2% | Heterozygous | 0,18 | | Normal | Concordant | |
| 10 | TP53 | c.524G>A | p.(Arg175His) | 1408 | 80,8% | Homozygous | -0,257 | Homozygous deletion | *TP53* | c.524G>A | p.(Arg175His) | 1386 | 38,8% | Heterozygous | -0,081 | | Normal | Discordant | |
| 10 | CDKN2A/B | NA | NA | NA | NA | NA | -0,901 | Homozygous deletion | *CDKN2A/B* | NA | NA | NA | NA | NA | -0,109 | | Normal | Discordant | |
| 11 | ERBB2 | NA | NA | NA | NA | NA | 3,273 | Amplification | *ERBB2* | NA | NA | NA | NA | NA | 3,791 | | Amplification | Concordant | |
| 11 | PIK3CA | c.3140A>G | p.(His1047Arg) | 737 | 41,4% | Heterozygous | 0,037 | Normal | *PIK3CA* | c.3140A>G | p.His1047Arg | 582 | 50,2% | Heterozygous | 0,06 | | Normal | Concordant | |
| 11 | TP53 | c.423C>A | p.(Cys141*) | 154 | 42,2% | Homozygous | -0,366 | Homozygous deletion | *TP53* | c.423C>A | p.(Cys141*) | 130 | 75,4% | Homozygous | -0,537 | | Homozygous deletion | Concordant | |
| 12 | FGFR1 | NA | NA | NA | NA | NA | 2,941 | Amplification | *FGFR1* | NA | NA | NA | NA | NA | 2,581 | | Amplification | Concordant | |
| 13 | NRAS | c.181C>A | p.(Gln61Lys) | 888 | 9,2% | Heterozygous | 0,072 | Normal | *NRAS* | c.181C>A | p.(Gln61Lys) | 1131 | 15,5% | Heterozygous | -0,183 | | Normal | Concordant | |
| 13 | TP53 | c.818G>A | p.(Arg273His) | 1765 | 34,6% | Heterozygous | -0,006 | Normal | *TP53* | c.818G>A | p.(Arg273His) | 993 | 33,3% | Heterozygous | -0,444 | | Normal | Concordant | |
| 14 | TP53 | c.472C>G | p.(Arg158Gly) | 1171 | 18,6% | Heterozygous | -0,021 | Normal | *TP53* | c.472C>G | p.(Arg158Gly) | 1305 | 78,3% | Homozygous | 0,346 | | Normal | Concordant | |
| 14 | STK11 | c.890G>A | p.(Arg297Lys) | 1114 | 29,8% | Non relevant | -0,456 | Non relevant | *STK11* | c.890G>A | p.(Arg297Lys) | 997 | 66,8% | Non relevant | -0,884 | | Non relevant | Concordant | |
| 15 | KRAS | c.35G>A | p.(Gly12Asp) | 1121 | 49,3% | Heterozygous | -0,173 | Normal | *KRAS* | c.35G>A | p.(Gly12Asp) | 1455 | 43,20% | Heterozygous | -0,005 | | Normal | Concordant | |
| 15 | PIK3CA | c.1634A>C | p.(Glu545Ala) | 2813 | 25,3% | Heterozygous | -0,136 | Normal | *PIK3CA* | c.1634A>C | p.(Glu545Ala) | 2728 | 35,00% | Heterozygous | -0,088 | | Normal | Concordant | |
| 15 | TP53 | c.524G>A | p.(Arg175His) | 696 | 32,2% | Heterozygous | 0,084 | Normal | *TP53* | c.524G>A | p.(Arg175His) | 2000 | 80,10% | Heterozygous | 0,061 | | Normal | Concordant | |
| 16 | KRAS | c.34G>C | p.(Gly12Arg) | 1909 | 53,3% | Heterozygous | 0,763 | Gain | *KRAS* | c.34G>C | p.(Gly12Arg) | 808 | 35,9% | Heterozygous | 0,693 | | Gain | Concordant | |
| 16 | TP53 | c.818G>T | p.(Arg273Leu) | 2798 | 55,3% | Heterozygous | -0,087 | Normal | *TP53* | c.818G>T | p.(Arg273Leu) | 1021 | 14,1% | Heterozygous | -0,001 | | Normal | Concordant | |
| 16 | CDKN2A/B | NA | NA | NA | NA | NA | -1,124 | Homozygous deletion | *CDKN2A/B* | NA | NA | NA | NA | NA | -0,669 | | Homozygous deletion | Discordant | |
| 17 | CCNE1 | NA | NA | NA | NA | NA | 1,063 | Amplification | *CCNE1* | NA | NA | NA | NA | NA | 1,752 | | Amplification | Concordant | |
| 17 | POLE | c.956A>G | p.(Asp319Gly) | 662 | 41,1% | Heterozygous | -0,033 | Normal | *POLE* | c.956A>G | p.(Asp319Gly) | 456 | 38% | Heterozygous | 0,008 | | Normal | Concordant | |
| 17 | TP53 | c.734 G>A | p.(Gly245Asp) | 1460 | 10,3% | Heterozygous | -0,083 | Normal | *TP53* | c.734 G>A | p.(Gly245Asp) | 664 | 32% | Heterozygous | -0,19 | | Normal | Concordant | |
| 18 | ESR1 | c.1138G>C | p.(Glu380Gln) | 2860 | 37,1% | Heterozygous | -0,012 | Normal | *ESR1* | c.1138G>C | p.(Glu380Gln) | 416 | 44% | Heterozygous | -0,017 | | Normal | Concordant | |
| 18 | PIK3CA | c.3140A>G | p.(His1047Arg) | 4040 | 32,9% | Heterozygous | -0,037 | Normal | *PIK3CA* | c.3140A>G | p.(His1047Arg) | 528 | 41% | Heterozygous | -0,081 | | Normal | Concordant | |
| 19 | CDKN2A | NA | NA | NA | NA | NA | -0,607 | Homozygous deletion | *CDKN2A* | NA | NA | NA | NA | NA | -2,168 | | Homozygous deletion | Discordant | |
| 19 | PIK3CA | NA | NA | NA | NA | NA | 1,689 | Amplification | *PIK3CA* | NA | NA | NA | NA | NA | 1,944 | | Amplification | Concordant | |
| 19 | TP53 | c.817C>T | p.(Arg273Cys) | 1271 | 23,7% | Heterozygous | 0,079 | Normal | *TP53* | c.817C>T | p.(Arg273Cys) | 498 | 43% | Heterozygous | 0,259 | | Normal | Concordant | |
| 19 | TP53 | c.36_49del | p.(Pro13Asnfs*11) | 2062 | 24,9% | Heterozygous | 0,079 | Normal | *TP53* | c.36_49del | p.(Pro13Asnfs*11) | 612 | 37% | Heterozygous | 0,259 | | Normal | Concordant | |
| 19 | NF1 | c.2484G>T | p.(Leu828Phe) | 840 | 15,8% | Heterozygous | 0,072 | Normal | *NF1* | c.2484G>T | p.(Leu828Phe) | 185 | 23,2% | Homozygous | -0,16 | | Homozygous deletion | Discordant | |
| 20 | PIK3CA | c.1624G>A | p.(Glu542Lys) | 4824 | 67,6% | Homozygous | -0,094 | Normal | *PIK3CA* | c.1624G>A | p.(Glu542Lys) | 3631 | 54% | Heterozygous | -0,026 | | Normal | Concordant | |
| 21 | CDK4 | NA | NA | NA | NA | NA | 0,612 | Gain | *CDK4* | NA | NA | NA | NA | NA | -0,084 | | Normal | Discordant | |
| 21 | MDM2 | NA | NA | NA | NA | NA | 0,612 | Gain | *MDM2* | NA | NA | NA | NA | NA | -0,084 | | Normal | Discordant | |
| 22 | STK11 | NA | NA | NA | NA | NA | -2,436 | Homozygous deletion | *STK11* | NA | NA | NA | NA | NA | -4,107 | | Homozygous deletion | Concordant | |
| 22 | TP53 | c.773A>C | p.(Glu258Ala) | 5196 | 84,4% | Homozygous | 0,268 | Normal | *TP53* | c.773A>C | p.(Glu258Ala) | 5567 | 95% | Homozygous | 0,289 | | Normal | Concordant | |
| 23 | NA | NA | NA | NA | NA | NA | NA | NA | *NA* | NA | NA | NA | NA | NA | NA | | NA | Concordant | |
| 24 | TP53 | c.844C>T | p.(Arg282Trp) | 990 | 70,1% | Homozygous | -0,047 | Normal | *TP53* | c.844C>T | p.(Arg282Trp) | 602 | 90,70% | Homozygous | 0,054 | | Normal | Concordant | |
| 25 | CCND1 | NA | NA | NA | NA | NA | 2,784 | Amplification | *CCND1* | NA | NA | NA | NA | NA | 3,026 | | Amplification | Concordant | |
| 25 | FGFR1 | NA | NA | NA | NA | NA | 2,849 | Amplification | *FGFR1* | NA | NA | NA | NA | NA | 2,811 | | Amplification | Concordant | |
| 26 | CCND1 | NA | NA | NA | NA | NA | 2,869 | Amplification | *CCND1* | NA | NA | NA | NA | NA | 2,889 | | Amplification | Concordant | |
| 26 | EGFR | NA | NA | NA | NA | NA | 3,487 | Amplification | *EGFR* | NA | NA | NA | NA | NA | 3,78 | | Amplification | Concordant | |
| 27 | PTEN | NA | NA | NA | NA | NA | -0,057 | Normal | *PTEN* | NA | NA | NA | NA | NA | -2,636 | | Homozygous deletion | Discordant | |
| 28 | PIK3CA | c.1633G>A | p.(Glu545Lys) | 2623 | 42,7% | Heterozygous | 0,102 | Normal | *PIK3CA* | c.1633G>A | p.(Glu545Lys) | 1926 | 65% | Heterozygous | 0,022 | | Normal | Concordant | |
| 29 | ESR1 | c.1138G>C | p.(Glu380Gln) | 3903 | 50,9% | Heterozygous | 1,116 | Gain | *ESR1* | c.1138G>C | p.(Glu380Gln) | 4062 | 50% | Heterozygous | 0,888 | | Gain | Concordant | |
| 29 | MAP2K4 | NA | NA | NA | NA | NA | -2,792 | Homozygous deletion | *MAP2K4* | NA | NA | NA | NA | NA | -1,409 | | Homozygous deletion | Concordant | |
| 29 | TP53 | c.277delC | p.(Leu93Cysfs*30) | 957 | 99,6% | Homozygous | -0,284 | Normal | *TP53* | c.277delC | p.(Leu93Cysfs*30) | 842 | 95% | Homozygous | -0,074 | | Normal | Concordant | |
| 30 | TP53 | c.405C>G | p.(Cys135Trp) | 930 | 54,0% | Heterozygous | -0,407 | Homozygous deletion | *TP53* | c.405C>G | p.(Cys135Trp) | 4739 | 53,7% | Heterozygous | -0,339 | | Homozygous deletion | Concordant | |
| 30 | KRAS | c.35G>A | p.(Gly12Asp) | 1137 | 25,5% | Heterozygous | 0,29 | Gain | *KRAS* | c.35G>A | p.(Gly12Asp) | 769 | 17,8% | Heterozygous | 0,148 | | Gain | Concordant | |
| 30 | CDKN2A | c.35_83del | p.(Ser12Cysfs*25) | 178 | 51,7% | Heterozygous | -0,438 | Homozygous deletion | *CDKN2A* | c.35_83del | p.(Ser12CysfsTer25) | 690 | 53,9% | Heterozygous | -0,369 | | Homozygous deletion | Concordant | |
| 30 | MAP2K4 | NA | NA | NA | NA | NA | -1,392 | Homozygous deletion | *MAP2K4* | NA | NA | NA | NA | NA | -1,281 | | Homozygous deletion | Concordant | |
| 31 | SUFU | c.597G>A | p.(=) | 308 | 63,0% | Heterozygous | -0,475 | Homozygous deletion | *SUFU* | c.597G>A | p.(=) | 354 | 10,7% | Heterozygous | -0,225 | | Normal | Discordant | |
| 32 | TP53 | c.360delG | p.(Lys120Asnfs*3) | 419 | 0,0% | Absent | -0,384 | Normal | *TP53* | c.360delG | p.(Lys120Asnfs*3) | 381 | 17,1% | Heterozygous | 0,096 | | Normal | Discordant | |
| 32 | NF1 | c.3859T>C | p.(Phe1287Leu) | 3255 | 55,6% | Heterozygous | -0,088 | Normal | *NF1* | c.3859T>C | p.(Phe1287Leu) | 2135 | 48,4% | Heterozygous | -0,122 | | Normal | Concordant | |
| 33 | TP53 | c.586C>T | p.(Arg196*) | 380 | 67,1% | Homozygous | 0,076 | Normal | *TP53* | c.586C>T | p.(Arg196Ter) | 166 | 0,4096 | Heterozygous | -0,278 | | Normal | Concordant | |
| 33 | KRAS | c.183A>C | p.(Gln61His) | 3477 | 49,0% | Heterozygous | -0,116 | Normal | *KRAS* | c.183A>C | p.(Gln61His) | 2554 | 0,1942 | Heterozygous | -0,219 | | Normal | Concordant | |
| 33 | CCND1 | NA | NA | NA | NA | NA | 5,518 | Amplification | *CCND1* | NA | NA | NA | NA | NA | 4,686 | | Amplification | Concordant | |
| 34 | TP53 | c.166G>T | p.(Glu56*) | 176 | 29,0% | Heterozygous | -0,147 | Normal | *TP53* | c.166G>T | p.(Glu56*) | 0 | 0,0% | Homozygous | 0,113 | | Normal | Concordant | |
| 34 | CDKN2A/2B | NA | NA | NA | NA | NA | -1,31 | Homozygous deletion | *CDKN2A/2B* | NA | NA | NA | NA | NA | -2,348 | | Homozygous deletion | Concordant | |
| 35 | ESR1 | c.1613A>G | p.(Asp538Gly) | 29 | 0,0% | Heterozygous | -0,694 | Normal | *ESR1* | c.1613A>G | p.(Asp538Gly) | 8 | 0% | Homozygous | -0,724 | | Homozygous deletion | Concordant | |
| 36 | PIK3CA | c.1633G>A | p.(Glu545Lys) | 3421 | 22,0% | Heterozygous | 0,032 | Normal | *PIK3CA* | c.1633G>A | p.(Glu545Lys) | 3856 | 59,1% | Heterozygous | -0,086 | | Normal | Concordant | |
| 36 | IDH1 | c.394C>T | p.(Arg132Cys) | 1817 | 16,6% | Heterozygous | 0,078 | Normal | *IDH1* | c.394C>T | p.(Arg132Cys) | 2392 | 45,9% | Heterozygous | 0,227 | | Normal | Concordant | |
| 36 | ALK | c.4723C>T | p.(Arg1575Cys) | 1622 | 13,1% | Heterozygous | 0,078 | Normal | *ALK* | c.4723C>T | p.(Arg1575Cys) | 1930 | 36,4% | Heterozygous | 0,227 | | Normal | Concordant | |
| 36 | MAP2K4 | c.719A>G | p.(Asp240Gly) | 4132 | 47,9% | Heterozygous | 0,192 | Normal | *MAP2K4* | c.719A>G | p.(Asp240Gly) | 7721 | 92,9% | Homozygous | 0,334 | | Gain | Concordant | |
| 36 | TP53 | c.817C>T | p.(Arg273Cys) | 2222 | 42,7% | Heterozygous | 0,192 | Normal | *TP53* | c.817C>T | p.(Arg273Cys) | 3328 | 92,3% | Homozygous | 0,229 | | Gain | Concordant | |
| 37 | TP53 | c.337_338del | p.(Phe113Leufs*35) | 977 | 15,2% | Heterozygous | 0,072 | Normal | *PTCH1* | c.1855G>A | p.(Val619Ile) | 724 | 26,4% | Heterozygous | 0,125 | | Normal | Concordant | |
| 38 | ESR1 | c.1613A>G | p.(Asp538Gly) | 149 | 21,5% | Heterozygous | 0,182 | Normal | *ESR1* | c.1613A>G | p.(Asp538Gly) | 145 | 0,3931 | Heterozygous | 0,53 | | Normal | Concordant | |
| 38 | FGFR1 | NA | NA | NA | NA | NA | 2,414 | Amplification | *FGFR1* | NA | NA | NA | NA | NA | 3,07 | | Amplification | Concordant | |
| 39 | NA | NA | NA | NA | NA | NA | NA | NA | *TP53* | c.158G>A | p.(Trp53*) | 150 | 14,0% | Heterozygous | -0,106 | | Normal | Discordant | |
| 40 | TP53 | c.517G>T | p.(Val173Leu) | 475 | 57,47 | Homozygous | 0,078 | Normal | *TP53* | c.517G>T | p.(Val173Leu) | 396 | 64,39 | Homozygous | 0,104 | | Normal | Concordant | |
| 40 | HRAS | c.37G>C | p.(Gly13Arg) | 238 | 18,91 | Heterozygous | 0,471 | Gain | *HRAS* | c.37G>C | p.(Gly13Arg) | 198 | 19,19 | Heterozygous | 0,592 | | Gain | Concordant | |
| 41 | NA | NA | NA | NA | NA | NA | NA | NA | *NA* | NA | NA | NA | NA | NA | NA | | NA | Concordant | |
| 42 | TP53 | c.96+1G>T | p.? | 1834 | 94,8% | Homozygous | 0,081 | Normal | *TP53* | c.96+1G>T | p.? | 854 | 96,8% | Homozygous | 0,006 | | Normal | Concordant | |
| 43 | NI | NI | NI | NI | NI | NI | NI | NI | *TP53* | c.376-1G>C | p.? | 654 | 99% | Homozygous | -0,203 | | Normal | Discordant | |
| 43 | NI | NI | NI | NI | NI | NI | NI | NI | *EGFR* | NA | NA | NA | NA | NA | 2,225 | | Amplification | Discordant | |
| 44 | MAP2K4 | c.846+2T>G | p.? | 2557 | 45,6% | Homozygous | -0,606 | Homozygous deletion | *MAP2K4* | c.846+2T>G | p.? | 3064 | 75,6% | Homozygous | -0,86 | | Homozygous deletion | Concordant | |
| 45 | NA | NA | NA | NA | NA | NA | NA | NA | *NA* | NA | NA | NA | NA | NA | NA | | NA | Concordant | |
| 46 | PIK3R1 | c.1708_1716del | p.(Ile571_Leu573del) | 2781 | 71,0% | Homozygous | -0,092 | Normal | *PIK3R1* | c.1708_1716del | p.(Ile571_Leu573del) | 5410 | 6,70% | Heterozygous | -0,011 | | Normal | Discordant | |
| 46 | KRAS | c.35G>A | p.(Gly12Asp) | 1637 | 56,1% | Heterozygous | 0,711 | Gain | *KRAS* | c.35G>A | p.(Gly12Asp) | 1499 | 8,10% | Heterozygous | 0,226 | | Normal | Concordant | |
| 46 | TP53 | c.586C>T | p.(Arg196Ter) | 76 | 93,4% | Homozygous | 0,056 | Normal | *TP53* | c.586C>T | p.(Arg196Ter) | 184 | 0 | Absent | 0,157 | | Normal | Discordant | |
| 46 | PTEN | NA | NA | NA | NA | NA | -0,93 | Homozygous deletion | *PTEN* | NA | NA | NA | NA | NA | 0,132 | | Normal | Discordant | |
| 47 | TP53 | c.839G>A | p.(Arg280Lys) | 560 | 53% | Heterozygous | -0,003 | Normal | *TP53* | c.839G>A | p.(Arg280Lys) | 800 | 70% | Homozygous | -0,023 | | Normal | Discordant | |
| 47 | MYC | NA | NA | NA | NA | NA | 2,672 | Amplification | *MYC* | NA | NA | NA | NA | NA | 3,389 | | Amplification | Concordant | |
| 48 | STK11 | c.374+1A>G | p.? | 1186 | 60,8% | Homozygous | -0,182 | Normal | *STK11* | c.374+1A>G | p.? | 3747 | 82,8% | Homozygous | -0,054 | | Normal | Concordant | |
| 48 | TP53 | c.742C>T | p.(Arg248Trp) | 6539 | 65,2% | Homozygous | -0,199 | Normal | *TP53* | c.742C>T | p.(Arg248Trp) | 14588 | 85,5% | Homozygous | -0,28 | | Normal | Concordant | |
| 48 | MYC | NA | NA | NA | NA | NA | 2,913 | Amplification | *MYC* | NA | NA | NA | NA | NA | 3,118 | | Amplification | Concordant | |
| 49 | TP53 | c.376-2A>G | p.? | 2518 | 45,2% | Heterozygous | -0,387 | Normal | *TP53* | c.376-2A>G | p.? | 1743 | 17,8% | Heterozygous | -0,014 | | Normal | Concordant | |
| 49 | MAP2K4 | NA | NA | NA | NA | NA | -1,067 | Homozygous deletion | *MAP2K4* | NA | NA | NA | NA | NA | -0,726 | | Homozygous deletion | Discordant | |
| 49 | ERBB2 | NA | NA | NA | NA | NA | 3,711 | Amplification | *ERBB2* | NA | NA | NA | NA | NA | 2,784 | | Amplification | Concordant | |
| 50 | ESR1 | c.1610A>C | p.(Tyr537Ser) | 310 | 20,7% | Heterozygous | -0,409 | Homozygous deletion | *ESR1* | c.1610A>C | p.(Tyr537Ser) | 424 | 0 | Absent | -0,094 | | Normal | Discordant | |
| 50 | PIK3CA | c.3140A>G | p.(His1047Arg) | 2607 | 32,4% | Heterozygous | -0,073 | Normal | *PIK3CA* | c.3140A>G | p.(His1047Arg) | 2923 | 0 | Absent | -0,126 | | Normal | Discordant | |
| 51 | TP53 | c.560-2delA | p.? | 320 | 67,8% | Homozygous | -0,09 | Normal | *TP53* | c.560-2delA | p.? | 1329 | 93,30% | Homozygous | 0,04 | | Normal | Concordant | |
| 52 | ATRX | c.5272+2T>G | p.? | 3124 | 18,6% | Heterozygous | 0,237 | Normal | *ATRX* | c.5272+2T>G | p.? | 3270 | 25,10% | Heterozygous | 0,339 | | Normal | Concordant | |
| 52 | ESR1 | c.1610A>C | p.(Tyr537Ser) | 165 | 12,7% | Heterozygous | -0,158 | Normal | *ESR1* | c.1610A>C | p.(Tyr537Ser) | 135 | 27,40% | Heterozygous | -0,318 | | Homozygous deletion | Concordant | |
| 52 | CCND1 | NA | NA | NA | NA | NA | 1,893 | Amplification | *CCND1* | NA | NA | NA | NA | NA | 2,613 | | Amplification | Concordant | |
| 53 | ESR1 | c.1613A>G | p.(Asp538Gly) | 388 | 20,4% | Heterozygous | 0,1 | Normal | *ESR1* | c.1613A>G | p.(Asp538Gly) | 264 | 15,9% | Heterozygous | 0,282 | | Normal | Concordant | |
| 53 | KRAS | c.35G>T | p.(Gly12Val) | 1010 | 43,9% | Heterozygous | -0,041 | Normal | *KRAS* | c.35G>T | p.(Gly12Val) | 893 | 49,2% | Heterozygous | -0,096 | | Normal | Concordant | |
| 53 | PIK3CA | c.3132T>A | p.(Asn1044Lys) | 4296 | 52,0% | Heterozygous | 0,017 | Normal | *PIK3CA* | c.3132T>A | p.(Asn1044Lys) | 3244 | 55,8% | Heterozygous | -0,057 | | Normal | Concordant | |
| 53 | CCND1 | NA | NA | NA | NA | NA | 1,991 | Amplification | *CCND1* | NA | NA | NA | NA | NA | 2,221 | | Amplification | Concordant | |
| 54 | KRAS | c.38G>A | p.(Gly13Asp) | 849 | 22,4% | Heterozygous | 0,089 | Normal | *KRAS* | c.38G>A | p.(Gly13Asp) | 1160 | 30,80% | Heterozygous | 0,096 | | Normal | Concordant | |
| 55 | PIK3CA | c.1636C>A | p.(Gln546Lys) | 5252 | 51,1% | Heterozygous | 0,319 | Normal | *PIK3CA* | c.1636C>A | p.(Gln546Lys) | 4695 | 31,1% | Heterozygous | 0,029 | | Normal | Concordant | |
| 55 | MAP2K4 | c.262A>T | p.(Arg88Ter) | 996 | 46,9% | Homozygous | -0,437 | Homozygous deletion | *MAP2K4* | c.262A>T | p.(Arg88Ter) | 1686 | 19,8% | Heterozygous | -0,075 | | Normal | Discordant | |
| 56 | BRAF | c.1799T>A | p.(Val600Glu) | 10113 | 46,0% | Heterozygous | 0,236 | Normal | *BRAF* | c.1799T>A | p.(Val600Glu) | 1893 | 33,50% | Heterozygous | 0,215 | | Normal | Concordant | |
| 57 | KRAS | c.35G>A | p.(Gly12Asp) | 104 | 34,6% | Heterozygous | 0,398 | Gain | *KRAS* | c.35G>A | p.(Gly12Asp) | 1860 | 40,90% | Heterozygous | 0,548 | | Gain | Concordant | |
| 57 | TP53 | c.376T>C | p.(Tyr126His) | 307 | 20,5% | Heterozygous | -0,017 | Normal | *TP53* | c.376T>C | p.(Tyr126His) | 408 | 36,50% | Heterozygous | 0,07 | | Normal | Concordant | |
| 57 | MYC | NA | NA | NA | NA | NA | 2,863 | Amplification | *MYC* | NA | NA | NA | NA | NA | 3,248 | | Amplification | Concordant | |
| 58 | ESR1 | c.1607T>A | p.(Leu536His) | 271 | 12,6% | Heterozygous | -0,071 | Normal | *ESR1* | c.1607T>A | p.(Leu536His) | 296 | 14,90% | Heterozygous | 0,233 | | Normal | Concordant | |
| 58 | CDKN2A | NA | NA | NA | NA | NA | -2,209 | Homozygous deletion | *CDKN2A* | NA | NA | NA | NA | NA | -2,988 | | Homozygous deletion | Concordant | |
| 59 | KRAS | c.38G>A | p.(Gly13Asp) | 1036 | 48,0% | Heterozygous | -0,002 | Normal | *KRAS* | c.38G>A | p.(Gly13Asp) | 1430 | 41,10% | Heterozygous | -0,053 | | Normal | Concordant | |
| 59 | TP53 | c.535C>T | p.(His179Tyr) | 2281 | 28,6% | Heterozygous | 0,508 | Gain | *TP53* | c.535C>T | p.(His179Tyr) | 2741 | 26,60% | Heterozygous | 0,342 | | gain | Concordant | |
| 60 | TP53 | c.953del | p.(Pro318Glnfs*27) | 948 | 43,8% | Homozygous | -0,393 | Homozygous deletion | *TP53* | c.953del | p.(Pro318Glnfs*27) | 880 | 47,70% | Homozygous | -0,599 | | Homozygous deletion | Concordant | |
| 60 | TSC1 | NA | NA | NA | NA | NA | -3,934 | Homozygous deletion | *TSC1* | NA | NA | NA | NA | NA | -3,802 | | Homozygous deletion | Concordant | |
| 61 | CDKN2A/CDKN2B | NA | NA | NA | NA | NA | -0,515 | Homozygous deletion | *CDKN2A/CDKN2B* | NA | NA | NA | NA | NA | -0,638 | | Homozygous deletion | Concordant | |
